# Exercise mitigates Dapagliflozin-induced skeletal muscle atrophy in STZ-induced diabetic rats

**DOI:** 10.1186/s13098-023-01130-w

**Published:** 2023-07-12

**Authors:** Xudong Yang, Lifeng Wang, Liangzhi Zhang, Xia Zhai, Xiusheng Sheng, Helong Quan, Hengjun Lin

**Affiliations:** 1 College of Physical Education and Health Sciences, Zhejiang Normal University, Jinhua, Zhejiang China; 2 Exercise and Metabolism Research Center, Zhejiang Normal University, Jinhua, Zhejiang China; 3 Medical Molecular Biology Laboratory, School of Medicine, Jinhua Polytechnic, Jinhua, Zhejiang China; 4 School of Sports Science and Physical Education, Research Center of Sports and Health Science, Northeast Normal University, 5268 Renmin Street, Changchun, Jilin 130024 China; 5 Department of Colorectal anal surgery, Jinhua people’s hospital, 267 Danxi East Road, Jinhua, Zhejiang 321007 China

**Keywords:** Dapagliflozin, Exercise, Type 2 diabetes, SGLT2 inhibitors, Skeletal muscle atrophy, Protein metabolism

## Abstract

**Background:**

Sodium-glucose cotransporter 2 inhibitors (SGLT2i) are commonly used in the management of type 2 diabetes mellitus (T2DM) and have been found to worsen the reduction of skeletal muscle mass in individuals with T2DM. This study aims to examine the potential of exercise in mitigating the skeletal muscle atrophy induced by SGLT2i treatment.

**Methods:**

A rat model of T2DM (40 male Sprague-Dawley rats; T2DM induced by a combination of high-fat diet and streptozotocin) was used to examine the effects of six-week treatment with Dapagliflozin (DAPA, SGLT2i) in combination with either aerobic exercise (AE) or resistance training (RT) on skeletal muscle. T2DM-eligible rats were randomized into the T2DM control group (CON, n = 6), DAPA treatment group (DAPA, n = 6), DAPA combined with aerobic exercise intervention group (DAPA + AE, n = 6), and DAPA combined with resistance training intervention group (DAPA + RT, n = 6). To assess the morphological changes in skeletal muscle, myosin ATPase and HE staining were performed. mRNA expression levels of Atrogin-1, MuRF1, and Myostatin were determined using quantitative PCR. Furthermore, protein expression levels of AKT, p70S6K, mTOR, FoXO1/3A, NF-κB, and MuRF1 were examined through western blotting.

**Results:**

Both the administration of DAPA alone and the combined exercise intervention with DAPA resulted in significant reductions in blood glucose levels and body weight in rats. However, DAPA alone administration led to a decrease in skeletal muscle mass, whereas RT significantly increased skeletal muscle mass and muscle fiber cross-sectional area. The DAPA + RT group exhibited notable increases in both total protein levels and phosphorylation levels of AKT and p70S6K in skeletal muscle. Moreover, the DAPA, DAPA + AE, and DAPA + RT groups demonstrated downregulation of protein expression (FoXO1/3A) and mRNA levels (Atrogin-1, MuRF1, and Myostatin) associated with muscle atrophy.

**Conclusions:**

Our findings provide support for the notion that dapagliflozin may induce skeletal muscle atrophy through mechanisms unrelated to protein metabolism impairment in skeletal muscle, as it does not hinder protein metabolic pathways while reduces muscle atrophy-related genes. Additionally, our observations reveal that RT proves more effective than AE in enhancing skeletal muscle mass and muscle fiber cross-sectional area in rats with T2DM by stimulating protein anabolism within the skeletal muscle.

**Supplementary Information:**

The online version contains supplementary material available at 10.1186/s13098-023-01130-w.

## Introduction

Diabetes mellitus (DM) is a prevalent metabolic disorder worldwide, with type 2 diabetes mellitus (T2DM) accounting for approximately 90% of cases [1, 2]. Among the complications of T2DM, skeletal muscle atrophy is particularly common, especially among elderly patients [3, 4]. Reduced activation of the AKT-mammalian target of rapamycin (mTOR)-phosphoprotein 70 ribosomal protein S6 kinase (p70S6K) signaling pathway in skeletal muscle, caused by insulin resistance (IR) and insulin deficiency, leads to decreased protein synthesis [5–8]. Additionally, chronic inflammation, a hallmark of T2DM, contributes to the loss of skeletal muscle mass through excessive protein degradation [9–11]. This process involves various proteins (FoXO1/3A, NF-κB) and genes (MAFbx/Atrogin-1 and muscle RING-finger protein-1/MuRF1) regulated by the ubiquitin proteasome system (UPS) and autophagy-lysosome system (ALS) [12–14].

In response to the growing number of T2DM patients, significant progress has been made in the development of hypoglycemic medications. Sodium-glucose cotransporter 2 inhibitors (SGLT2i), for instance, directly reduce glucose uptake in the proximal renal tubules, resulting in a hypoglycemic effect and subsequent weight loss [15]. The metabolic effects indirectly induced by SGLT2i are closely associated with weight loss, primarily through a reduction in adipose tissue. This decrease in adipose tissue leads to a decline in the production of pro-inflammatory cytokines, thereby mitigating systemic inflammation and improving IR [16, 17]. Numerous studies have reported that weight loss often accompanies a decrease in lean body mass, primarily due to skeletal muscle mass reduction [18–20]. Patients treated with SGLT2i have shown decreased skeletal muscle mass and strength, as well as varying degrees of myopathy [18, 19]. Consequently, there is a concern that SGLT2i may increase the risk of muscle atrophy.

In patients receiving SGLT2i treatment, regular exercise has proven to be an effective non-pharmacological complement, enhancing glycemic control and leading to significant reductions in body fat levels. Specifically, combining SGLT2i treatment with aerobic exercise (AE) has demonstrated superior effectiveness in lowering blood glucose levels, improving insulin sensitivity and exercise capacity, and significantly reducing adipose tissue accumulation [21, 22]. However, the potential synergistic effects of SGLT2i combined with AE on enhancing skeletal muscle mass have not been well-established. According to the collaborative guidelines of the American College of Sports Medicine and the American Diabetes Association, individuals with T2DM are advised to engage in a minimum of 2–3 resistance training (RT) sessions per week, alongside AE, with moderate-to-heavy intensity [23]. RT, as a non-pharmacological intervention, has shown efficacy in improving skeletal muscle atrophy in various diseases, including diabetes, Alzheimer’s disease, and cachexia [24–26]. This effect can be primarily attributed to the increased activation of proteins involved in the protein synthesis pathway, particularly AKT-mTOR-p70S6K, in skeletal muscle [25, 26].

The aim of this study is to evaluate the impact of SGLT2i on skeletal muscle mass in rats with T2DM, and to explore the potential protective effect of exercise against muscle mass loss induced by SGLT2i. Furthermore, this study seeks to elucidate the underlying mechanism behind this phenomenon.

## Methods

### Animals and induction of T2DM

Forty male Sprague-Dawley rats of SPF grade, aged 4 weeks, were obtained from the Laboratory Animal Center at the Zhejiang Academy of Medical Sciences in Hangzhou, China. The rats were housed in the laboratory animal facility at Zhejiang Normal University (ZJNU) under controlled environmental conditions, including a constant temperature of 22 ± 2 °C, a humidity level of 55 ± 5%, and a 12-hour light/dark cycle. They were provided with ad libitum access to food and water. After a one-week acclimatization period, the rats were fed a high-fat diet comprising 35% of their total energy intake, obtained from Boaigang Biological Technology in Beijing, China. After four weeks on this diet, the rats underwent intraperitoneal injection of streptozotocin (STZ) at a dose of 35 mg/kg, dissolved in a sodium citrate buffer (50 mM, pH 4.5), following a 5-hour fasting period. At 13 weeks, a fasting blood glucose test was performed, and rats with blood glucose levels exceeding the threshold of > 16.7 mmol/L were randomly assigned to one of four groups: T2DM control group (CON, n = 6), Dapagliflozin group (DAPA, n = 6), dapagliflozin combined with aerobic exercise group (DAPA + AE, n = 6), and dapagliflozin combined with resistance training group (DAPA + RT, n = 6). Following one week of adaptation, the Dapagliflozin administration and exercise interventions commenced in the 14th week and continued for six weeks. At the end of the six-week treatment period, the rats underwent a 14-hour fasting period before being euthanized using ethyl ether.

All experimental procedures strictly adhered to the rules, regulations, and operating procedures governing laboratory animals at ZJNU. The experimental protocol and exercise plan were approved by the Animal Welfare and Ethics Committee of ZJNU (ZSDW2022015).

### Dapagliflozin administration

The rats in the DAPA, DAPA + AE, and DAPA + RT groups received dapagliflozin (10 mg/kg/day) via oral gavage in ultrapure water between 17:00 and 19:00 each day. In contrast, the rats in the CON group were administered the same volume of 0.9% physiological saline solution at the same time, instead of dapagliflozin.

### Fasting blood glucose analysis

After a 14-hour fasting period, tail vein blood samples were collected to measure fasting blood glucose levels using a glucometer (Sinocare, Changsha, China).

### Resistance training and aerobic exercise protocol

In the DAPA + RT group, rats underwent resistance training in the form of tail load-bearing ladder climbing [27]. The ladder had dimensions of 1.1 × 0.18 m, with a 2 cm gap height and an inclination angle of 80–85°. The load-bearing device consisted of 50 mL tubes containing equal-weight steel balls. The tubes were attached to the proximal end of the rats’ tails using medical tape, and the load was adjusted by varying the number of steel balls. The ladder climbing exercise regimen consisted of 8–12 trials, with weights set at 50% (trials 1 and 2), 75% (trials 3 and 4), 90% (trials 5 and 6), and 100% (trials 7 and 8) of the rats’ body weight, which was updated weekly. This training program was repeated three times per week for a duration of six weeks.

In the DAPA + AE group, rats were familiarized with an animal treadmill (Zhenhua Biologic Apparatus Facilities, Anhui, China) through four sessions, each lasting between 10 and 20 m/min, before the formal exercise period. During the formal exercise period, the rats underwent continuous treadmill running for a total duration of 1 h at a pace of 25 m/min, corresponding to approximately 60–70% of their maximum oxygen uptake, and at a 0° incline.

### Muscle tissue extraction

The Soleus (Sol), Gastrocnemius (Gas), Flexor Hallucis Longus (FHL), and Extensor Digitorum Longus (EDL) muscles were extracted and weighed using a laboratory balance (Sartorius Balances, Gottingen, Germany). The weights were recorded while the muscles were still wet. For protein analysis, muscle tissues were rinsed with ice-cold saline and rapidly frozen in liquid nitrogen. They were then stored at − 80 °C using a deep freezer (Thermo Fisher Scientific, Waltham, USA). Muscle tissues designated for staining were embedded in an optimal cutting temperature compound (Sakura, Tokyo, Japan), frozen in isopentane (Macklin, C14950843, Shanghai, China), and either stored at − 80 °C or used immediately. Serial cross sections with a thickness of 8 μm were produced using a cryotome (Leica Microsystems, Nussloch, Germany).

### Western blot analysis

The skeletal muscle tissue (EDL) was homogenized using RIPA lysis buffer (Thermo, 89,900, IL, USA) supplemented with Halt™ Protease and Phosphatase Inhibitor Single-Use Cocktail (Thermo, 1,861,280, IL, USA). The resulting supernatant was collected, and the total protein content was measured using the Rapid Gold BCA Protein Assay Kit (Thermo Scientific, A53226, IL, USA) according to the manufacturer’s instructions. Proteins (25 µg) were separated by electrophoresis on sodium dodecyl sulfate-polyacrylamide gels ranging from 8 to 12%. The separated proteins were then transferred onto polyvinylidene difluoride membranes (Bio-Rad, 1,620,177, CA, USA) and blocked with 5% skimmed milk (Bio-Rad, 1,706,404, CA, USA) for 2 h. Afterward, the membranes were washed three times with TBST and incubated overnight at 4 °C with primary antibodies. The primary antibodies used in this study included AKT, p-AKT (Ser473), mTOR, p70S6K, p-p70S6K, and p-FOXO3A (all from Cell Signaling Technology, MA, USA; 1:1000), as well as FOXO3A, FOXO1, NF-κB, and MuRF1 (all from Proteintech, Wuhan, China; 1:1000). A secondary antibody, horseradish peroxidase (HRP)-conjugated goat anti-rabbit antibody (Cell Signaling Technology, Danvers, MA, USA; 1:2000), was applied. The protein bands were visualized using the ChemiDoc system (Bio-Rad, California, USA) and quantified using Image J software.

### Myosin ATPase and hematoxylin and eosin (HE) staining

The EDL muscle was subjected to myosin ATPase and HE staining following the instructions provided in the kit manual (Solarbio, Beijing, China). Subsequently, the tissue samples were observed using a light microscope (DM 2500; Leica Microsystems) after mounting the slides with resin glue. H&E and myosin ATPase staining images were obtained from six slides per group, each containing samples from six rats. The scale bars for the H&E images were set at 50 and 100 μm (magnification ×400 and ×200), with 50–200 myofibers counted per picture, while for the myosin ATPase staining images, the scale bars were set at 100 μm (magnification ×200), with 100–200 myofibers counted per picture. For manual quantification of muscle fiber cross-sectional area within a 400× field of view, the images were analyzed in ImageJ software. The freehand tool was used to encircle all individual intact myofibers, excluding incomplete myofibers at the image edges. The methodology for assessing muscle fiber cross-sections is described in detail in previous studies [28]. The counting function in ImageJ was employed to manually record the number of type I muscle fibers (dark) within a 200× field of view (image area: 2500 μm²). To minimize errors arising from manual handling, three individuals repeated these procedures independently.

### RNA isolation and real time quantitative PCR (RT-qPCR)

Total RNA extraction from the EDL muscle was carried out using TRIzol lysate (Ambion, Austin, Texas, USA). The concentration of RNA was determined using Nanodrop spectrophotometry (Thermo Fisher Scientific, Waltham, USA). The cDNA synthesis was performed using the PrimeScript™ RT Master Mix kit (Takara, Shiga, Japan) according to the manufacturer’s instructions. mRNA expression was measured using the real-time quantitative PCR (RT-qPCR) system (Thermo Fisher Scientific, Waltham, USA) with the SYBR™ Select Master Mix kit (Takara, Shiga, Japan). Gene-specific primers were used for amplification, including Myostatin (forward: 5′-GCTGTAACCTTCCCAGGACC-3′, reverse: 5′-AGTCCCATCCAAAGGCTTCG-3′), Atrogin-1 (forward: 5′-AGCTTGTGCGATGTTACCCA-3′, reverse: 5′-GGTGAAAGTGAGACGGAGCA-3′), MuRF1 (forward: 5′-ACCAAGGAAAACAGCCACCA-3′, reverse: 5′-GGCTGTTTCCACAAGCTTGG-3′), and GAPDH (forward: 5′-TGATGGGTGTGAACCACGAG-3′, reverse: 5′-AGTGATGGCATGGACTGTGG-3′). The relative expression levels of each target gene were analyzed using the ΔΔCt method and normalized to GAPDH as an endogenous control.

### Statistical analyses

The data were presented as mean ± SEM. Paired samples t-tests and one-way ANOVA were employed to determine the significance of differences, followed by Tukey post hoc tests. Statistical significance was defined as a p-value < 0.05. GraphPad Prism software version 9 was used to conduct all statistical analyses.

## Results

### Weights of body, subcutaneous fat, and skeletal muscle for each group

At the beginning of the experiment, there were no significant differences in the initial body weights of the rats in each group (refer to Table 1). Following 6 weeks of treatment, the DAPA group (-3.3% weight), DAPA + AE group (-4% weight), and DAPA + RT group (-2% weight) showed significantly reduced body weights compared to the CON group (P < 0.05, Table 1). Notably, both the DAPA group (+ 20.8% weight) and DAPA + RT group (+ 22.7% weight) exhibited a significant reduction in body weight gain compared to the CON group (+ 25% weight) (P < 0.05, Table 1), while the DAPA + AE group demonstrated an even more pronounced reduction (+ 19.5% weight) (P < 0.01, Table 1). The DAPA group (-29.2% weight) and DAPA + RT group (–28.2% weight) also showed a significant decrease in subcutaneous fat wet weight compared to the CON group (P < 0.01), with the DAPA + AE group displaying the most prominent reduction (–33.8% weight) (P < 0.001, Table 1). Furthermore, the loss of body weight was accompanied by a reduction in skeletal muscle mass. Specifically, the EDL muscle weight of the DAPA group significantly decreased (P < 0.05, Table 1) compared to the CON group (-9.3% weight) and DAPA + RT group (–8.6% weight). These findings suggest that RT was effective in preventing the loss of skeletal muscle mass induced by DAPA.

**Table 1:** Comparisons of body weight, subcutaneous fat weight and muscle weight in each group

	CON	DAPA	DAPA + AE	DAPA + RT
**Initial BW (g)**	480 ± 5.30	481 ± 6.30	483 ± 4.90	480 ± 7.80
**Final BW (g)**	601 ± 6.50	581 ± 9.60*	577 ± 5.60*	589 ± 11.70*
**Change in body weight (g)**	121 ± 12.80	102 ± 33.30*	94 ± 23.90**	104.5 ± 32.10*
**Subcutaneous fat weight (g)**	2.16 ± 0.42	1.53 ± 0.25**	1.43 ± 0.19***	1.55 ± 0.21**
**Sol muscle weight (mg)**	342.50 ± 10.70	332.50 ± 8.04	335.00 ± 8.16	335.83 ± 9.32
**FHL muscle weight (mg)**	516.67 ± 25.11	492.50 ± 40.08	507.50 ± 27.80	544.16 ± 37.46
**Gas muscle weight (mg)**	3155.00 ± 72.86	3039.17 ± 67.42	3065.00 ± 59.02	3104.58 ± 93.68
**EDL muscle weight (mg)**	285.37 ± 5.38^#^	258.87 ± 10.47	267.63 ± 10.47	283.33 ± 17.04^#^

The values are presented as means ± SEM. BW, body weight; Sol, soleus muscle; FHL, flexor hallucis longus muscle; Gas, gastrocnemius muscle; EDL, extensor digitorum longus muscle. Statistical difference from the CON group is denoted by (^*^P < 0.05; ^**^P < 0.01; ^***^P < 0.001), while statistical difference from the DAPA group is denoted by (^#^P < 0.05)

### Changes in fasting blood glucose, skeletal muscle relative mass, and muscle fiber morphology in each group

**Fig. 1:**
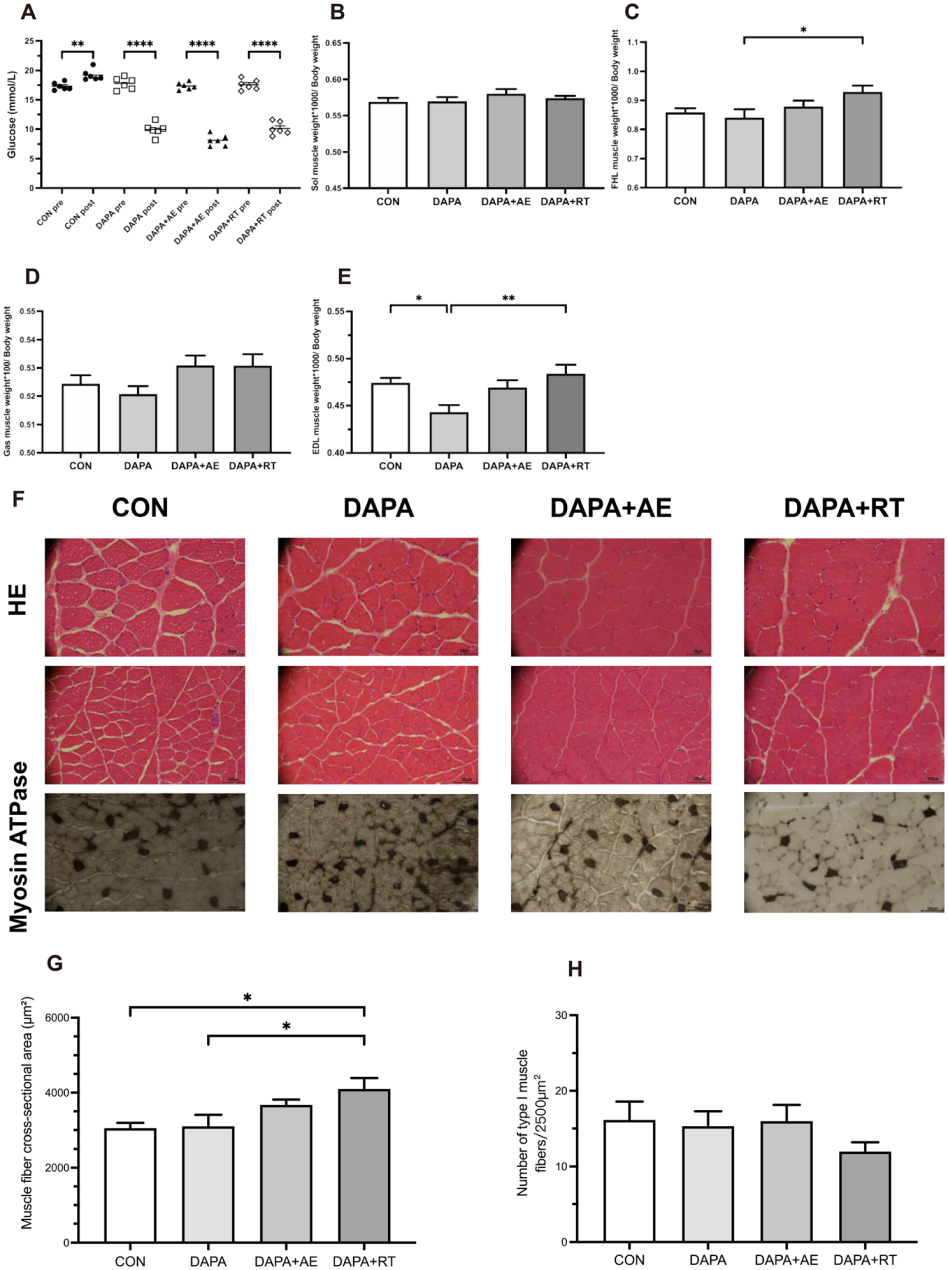
Effects of DAPA or DAPA combined with exercise on fasting blood glucose, skeletal muscle relative mass, and EDL muscle fiber morphology. (A) The changes in fasting blood glucose pre- and post-intervention. The ratios of the average muscle weights of the right and left Sol, FHL, Gas, and EDL muscles to body weight are shown in panels (B–E). HE and Myosin ATPase staining are shown in panel (F). These images were acquired from six slides per group of six rats at a scale of 50 μm (magnification ×400) and 100 μm (magnification ×200). (G) Myofiber cross-sectional areas were calculated using ImageJ software as the sum of individual myofiber areas within a 400× field of view divided by the total number of intact myofibers (excluding incomplete myofibers at the image edges). (H) The number of type I muscle fibers in each group was determined using ImageJ software. All data are shown as the mean ± SEM (n = 6 per group). Paired samples t-tests were performed for A, while the one-way ANOVA with Tukey post hoc test was utilized for B-H (*P < 0.05; **P < 0.01; ****P < 0.0001)

**Fig. 2:**
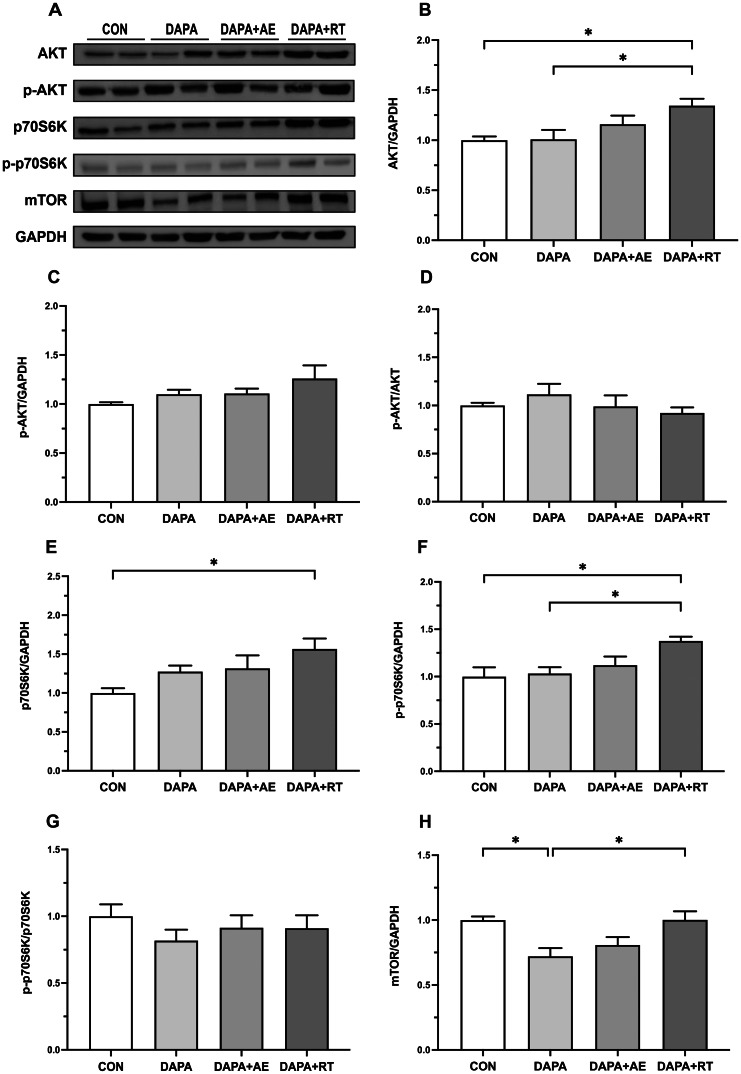
Effects of DAPA or DAPA combined with exercise on the expression of anabolic-related proteins in the extensor digitorum longus muscle of rats. The quantification of AKT, p70S6K, and mTOR proteins is shown in panels (A), (D), and (G), respectively. The phosphorylation levels of AKT and p70S6K proteins are shown in panels (B) and (E), respectively. The relative expressions of p-AKT and p-p70S6K are shown in panels (C) and (F). GAPDH was employed as an internal control. All data are shown as the mean ± SEM (n = 6 per group), and statistical analysis involved a one-way ANOVA test with Tukey’s post hoc evaluation (*P < 0.05)

Following a six-week treatment with DAPA or DAPA combined with exercise, T2DM rats exhibited a significant reduction in blood glucose levels (P < 0.0001, Fig. 1A). In contrast, there were no significant differences in the relative weight of Sol and Gas muscles among the four groups, an increasing trend was observed in the DAPA + AE and DAPA + RT groups (Fig. 2B, D). The relative weight of FHL muscle was significantly higher in the DAPA + RT group compared to the DAPA group (P < 0.05, Fig. 2C). Additionally, the relative weight of EDL muscle was significantly lower in the DAPA group compared to the CON group (P < 0.05, Fig. 1E), whereas it was significantly higher in the DAPA + RT group (P < 0.01, Fig. 1E). Notably, muscle fiber dispersion was observed in the CON and DAPA groups, but the DAPA + AE and DAPA + RT groups exhibited improvement (Fig. 1F), suggesting that exercise could partially alleviate the increase in muscle fiber dispersion caused by T2DM or SGLT2i. Furthermore, the DAPA + RT group showed a 34.5% and 32.4% increase in skeletal muscle cross-sectional area compared to the CON and DAPA groups, respectively (P < 0.05, Fig. 1G). The DAPA + AE group demonstrated a trend toward improvement, although without a significant increase (Fig. 1G). While there was no significant change in the number of type I muscle fibers in each group, there was a trend toward a decrease in the DAPA + RT group (Fig. 1H). These findings suggest that exercise can effectively mitigate DAPA-induced muscle mass loss and improve muscle morphology, with RT yielding a more pronounced effect.

### Expression of anabolic-related proteins AKT, p70S6K, and mTOR

The results depicting the expression levels of anabolic-related proteins in the EDL muscle following treatment with DAPA or DAPA combined with exercise are presented in Fig. 2. The findings reveal that AKT expression was significantly higher in the DAPA + RT group compared to the CON and DAPA groups (P < 0.05, Fig. 2B), while the levels of AKT phosphorylation and relative phosphorylation did not show significant variation among the groups (Fig. 2C, D). Both total and phosphorylation levels of p70S6K were elevated in the skeletal muscle of rats in the DAPA + RT group compared to the CON group (P < 0.05, Fig. 2E, F). Moreover, the phosphorylation levels of p70S6K were significantly higher in the DAPA + RT group compared to the DAPA group (P < 0.05, Fig. 2F), although there was no statistically significant difference in the relative phosphorylation levels between the groups (Fig. 2G). These findings suggest that exercise, particularly RT, can promote protein anabolism in skeletal muscle during SGLT2i treatment for T2DM. Additionally, mTOR expression levels were significantly reduced in the DAPA group compared to the CON and DAPA + RT groups (P < 0.05, Fig. 2H), indicating that DAPA affects mTOR expression in skeletal muscle, with RT acting to reverse this effect.

### Expression of muscle atrophy-related proteins: FOXOs, NF-κB, and MuRF1

**Fig. 3:**
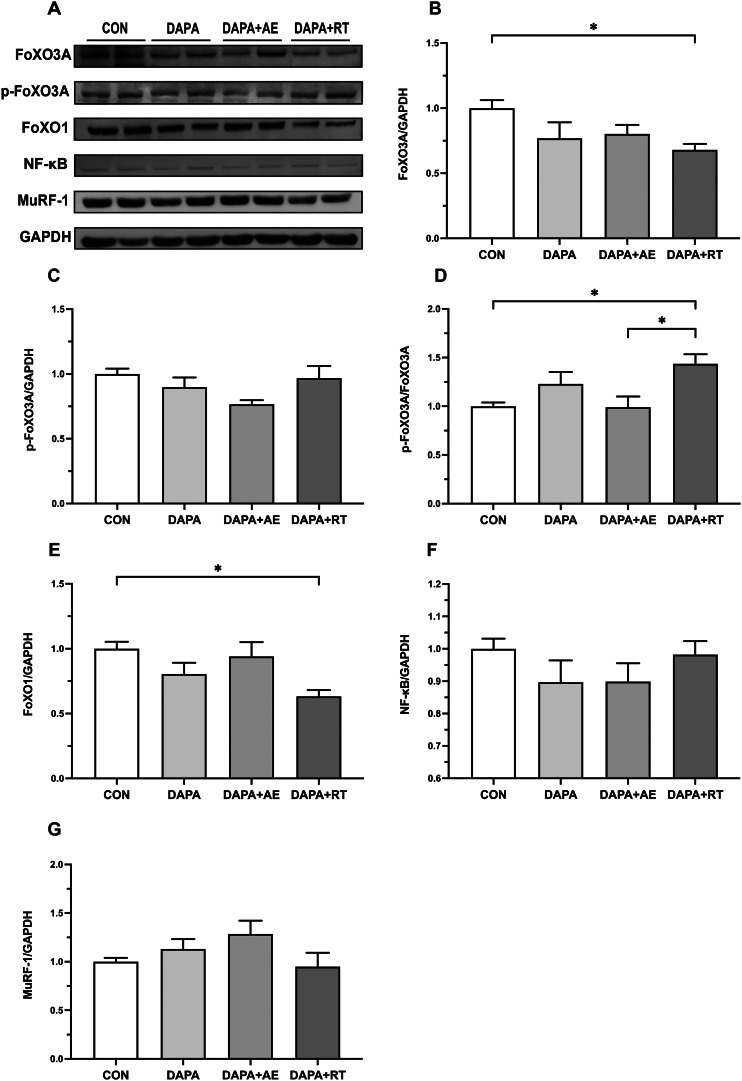
Effect of DAPA or DAPA combined with exercise on the expression of atrophy-associated proteins in the extensor digitorum longus muscle of rats. The quantification of FoXO3A, FoXO1, NF-κB, and MuRF1 proteins is depicted in panels (A), (D), (E), and (F), respectively. The phosphorylation levels of the FoXO3A protein are shown in panel (B), while the relative expression of p-FoXO3A protein is shown in panel (C). GAPDH was employed as an internal control. All data are shown as the mean ± SEM (n = 6 per group), and statistical analysis involved a one-way ANOVA test with Tukey’s post hoc evaluation (*P < 0.05)

Figure 3 presents the expression levels of atrophy-related proteins in the EDL muscle following treatment with DAPA or DAPA combined with exercise. In the DAPA + RT group, the expression of FoXO1 and 3 A proteins was significantly lower compared to the CON group (P < 0.05, Fig. 3B, E), with a decreasing trend observed in the DAPA and DAPA + AE groups. Additionally, the relative phosphorylation levels of FoXO3A in the skeletal muscle of DAPA + RT rats were significantly higher than those in the CON and DAPA + AE groups (P < 0.05, Fig. 3D), although no statistically significant difference was found in the phosphorylation levels of FoXO3A between the groups (Fig. 3C). These findings indicate that DAPA combined with RT resulted in a decrease in the total FoXO1/3A protein content in the skeletal muscle of T2DM rats while promoting FoXO3A phosphorylation. Furthermore, no significant difference in the protein expression of NF-κB and MuRF1 was observed among the four groups (Fig. 3F, G).

### Expression of muscle atrophy-related mRNA: Atrogin-1, MuRF1, and myostatin

**Fig. 4:**
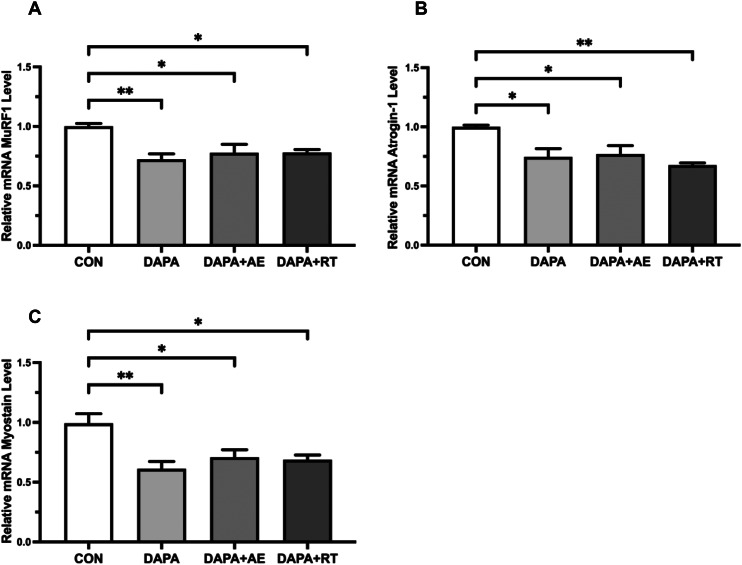
Effect of DAPA or DAPA combined with exercise on atrophy-related mRNA expression levels in EDL muscle of rats. The relative mRNA expression of Atrogin-1, MuRF1, and Myostatin is shown in panels (A-C). All data are shown as the mean ± SEM (n = 6 per group), and statistical analysis involved a one-way ANOVA test with Tukey’s post hoc evaluation (^*^P < 0.05)

Figure 4 illustrates the expression levels of atrophy-related mRNA in the EDL muscle following treatment with DAPA or DAPA combined with exercise. Evaluation of Atrogin-1 mRNA expression revealed a significant decrease in the skeletal muscle of rats in the DAPA and DAPA + AE groups compared to the CON group (P < 0.05, Fig. 4A), with a more pronounced decline observed in the DAPA + RT group (P < 0.01, Fig. 4A). Similarly, the mRNA expression of MuRF1 and Myostatin in the skeletal muscle of T2DM rats was significantly reduced in the DAPA + AE and DAPA + RT groups (P < 0.05, Fig. 4B, C), with a more substantial decrease occurring in the DAPA group (P < 0.01, Fig. 4B, C) when compared to the CON group. These findings indicate that both DAPA and DAPA combined with exercise effectively suppress the expression of atrophy-related mRNA in the skeletal muscle of T2DM rats.

## Discussion

The findings of our study revealed a decrease in skeletal muscle mass in rats with T2DM following SGLT2i administration. This aligns with previous studies in both humans and animals, which have consistently shown a correlation between SGLT2i and a reduction in skeletal muscle mass or lean body mass [17–19]. Although our study observed a significant decrease specifically in the EDL muscle mass, we attribute this to the relatively short duration of the intervention. Moreover, it seems that muscles with fast-twitch fibers, such as EDL and FHL, are more prone to atrophy in individuals with diabetes than slow-twitch fiber muscles like SOL [29, 30]. Additionally, SGLT2i could cause energy loss, which might result in the preferential use of proteins in fast-twitch muscles as a source of energy, therefore accelerating the loss of EDL [31]. We hypothesize that prolonged administration of SGLT2i may lead to more substantial loss of skeletal muscle mass. Notably, exercise mitigated the SGLT2i-induced decline in skeletal muscle mass and also enhanced muscle fiber cross-sectional area, with RT demonstrating a significant impact. These results are consistent with a study by JAE-HOON et al. [32], which highlighted the benefits of RT in improving skeletal muscle morphology. We propose that exercise can counteract the reduction in skeletal muscle mass caused by SGLT2i, with RT being particularly effective. However, further investigations are needed to determine the optimal exercise mode and frequency. The regulation of skeletal muscle mass is influenced by various pathways, including the insulin-like growth factor-1 (IGF-1)/insulin-AKT-mTOR pathway and the myostatin-Smad family transcription factors 2 and 3 pathway, which respectively promote or inhibit protein anabolism [33, 34]. The AKT-mTOR pathway is responsible for activating downstream target protein p70S6K, which plays a crucial role in promoting protein synthesis and muscle hypertrophy [35]. Insulin deficiency and IR disrupt the balance of protein metabolism in skeletal muscle, and RT can partially counteract the effects of these factors on muscle atrophy [36]. Studies on hypertrophy and atrophy have consistently demonstrated that RT induces muscle hypertrophy by upregulating the AKT-mTOR-p70S6K pathway [37, 38]. Interestingly, our study did not find a significant increase in the levels of AKT and p70S6K in rats treated with DAPA or DAPA combined with AE compared to the CON group. However, a trend toward an increase was observed, which may become more pronounced with longer treatment duration. Nonetheless, RT significantly upregulated the expression of AKT and p70S6K, indicating its potential to promote skeletal muscle protein anabolism during T2DM or SGLT2i treatment. Furthermore, our findings suggest that SGLT2i does not impede the effects of RT and may even have additional benefits in activating protein anabolic pathways. Notably, our study revealed a significant decrease in mTOR expression levels in skeletal muscle following DAPA treatment, which aligns with previous evidence demonstrating a similar decrease in mTOR expression levels in the liver [39]. However, further research is needed to confirm this observation due to the limited available evidence.

The inhibition of insulin or IGF-1 signaling pathway leads to reduced AKT activity and decreased phosphorylation of transcription factors, including FoxO family transcription factors 1 and 3 A [40, 41]. This ultimately results in increased protein degradation through the modulation of ALS and UPS [42]. Okamura et al. reported that 8 weeks of Luseogliflozin treatment reduced FoXO1 expression and increased muscle cross-sectional area in Db/Db mice with muscle atrophy [43]. Similarly, our study demonstrated that SGLT2i, both alone and in combination with exercise, reduced the expression levels of FoXO1/3A in skeletal muscle. Additionally, the combination of SGLT2i and RT significantly increased the relative phosphorylation levels of FoXO3A, indicating that RT can prevent protein degradation by promoting the AKT-FoXO3A pathway.

Furthermore, several genes involved in protein degradation, collectively known as “atrophy genes” [40, 44], are implicated in muscle atrophy. These genes include muscle RING finger 1 (MuRF1 or TRIM63) and Atrogin-1 (also known as MAFbx or FBXO32), which are muscle-specific E3 ubiquitin ligases activated by FOXOs and play a crucial role in inducing muscle atrophy [45]. Recent studies have shown that SGLT2i improve systemic inflammation by reducing adipose tissue content and downregulating the expression of Atrogin-1 and MuRF1 genes in skeletal muscle [46, 47]. Our findings support the notion that DAPA and DAPA combined with exercise reduce subcutaneous fat accumulation and significantly downregulate mRNA expression of Atrogin-1 and MuRF1 in skeletal muscle. Although the protein expression levels of MuRF1 did not differ in our study, Bamba et al. observed a reduction in the intensity of MuRF1 expression in FHL muscle following DAPA treatment [47]. These discrepancies in results may be attributed to variations in skeletal muscle type, animal models, or interventions, highlighting the need for further studies to validate these findings. Furthermore, Myostatin serves as a negative regulator of protein synthesis in skeletal muscle [48]. The inhibitory effect of the Myostatin-Smad pathway on the AKT-mTOR pathway activation results in reduced protein synthesis, promoting protein degradation and muscle mass loss [49, 50]. Our findings demonstrate that DAPA or DAPA combined with exercise significantly reduces the mRNA expression level of Myostatin, providing further support for the observations by Bamba et al. that SGLT2i downregulates transcripts associated with muscle atrophy [47].

In summary, our analysis suggests that the SGLT2i-induced skeletal muscle loss is not a result of impairment in muscle protein metabolism pathways, as DAPA suppressed the expression of genes associated with muscle atrophy and did not hinder protein anabolic pathways. Instead, it is likely attributed to the significant energy loss of 52–85 g of glucose per day (200–300 kcal/day) caused by SGLT2i treatment, leading to weight loss and increased substrate metabolism [15, 51]. Previous studies have demonstrated that DAPA promotes fat oxidation and mitochondrial metabolism, similar to caloric restriction [52], and enhances the availability of muscle-derived amino acids for hepatic gluconeogenesis [31]. These findings support our hypothesis that the caloric loss induced by SGLT2i may accelerate the breakdown of skeletal muscle for energy substrates, resulting in muscle mass loss. Exercise effectively mitigates DAPA-induced skeletal muscle atrophy, including changes in muscle mass and morphology, primarily through the up-regulation of skeletal muscle protein anabolic pathways.

## Conclusion

Our study provides evidence that short-term use of SGLT2i may result in skeletal muscle mass reduction, and long-term administration could potentially increase the risk of muscle atrophy. However, engaging in exercise, specifically RT, can counteract these effects. Importantly, we found that SGLT2i does not impair protein metabolism in skeletal muscle, and instead, the activation of pathways related to muscle atrophy is downregulated. This suggests that mechanisms other than impaired protein metabolism, such as the significant loss of urinary sugar leading to caloric deprivation, are involved in the SGLT2i-induced skeletal muscle loss.

### Electronic supplementary material

Below is the link to the electronic supplementary material.

Supplementary Material 1

## Data Availability

The datasets used and/or analyzed during the current study are available from the corresponding author on reasonable request.

## Abbreviations

SGLT2i: Sodium-glucose transporter 2 inhibiters
T2DM: Type 2 diabetes mellitus
DAPA: Dapagliflozin
AE: Aerobic exercise
RT: Resistance training
P70S6K: Phosphoprotein 70 ribosomal protein S6 kinase
Atrogin-1: Muscle atrophy F-box
MuRF1: Muscle RING finger 1
FoxOs: Forkhead Box O family transcription factors
IR: Insulin resistance
AKT: Protein kinase B
mTOR: Mechanical target of rapamycin
UPS: Ubiquitin-proteasome system
ALS: Autophagy-lysosome system
NF-κB: Nuclear factor-kappa B
