# Correction: Exercise mitigates Dapagliflozin-induced skeletal muscle atrophy in STZ-induced diabetic rats

**DOI:** 10.1186/s13098-023-01139-1

**Published:** 2023-08-17

**Authors:** Xudong Yang, Lifeng Wang, Liangzhi Zhang, Xia Zhai, Xiusheng Sheng, Hengjun Lin, Helong Quan

**Affiliations:** 1 College of Physical Education and Health Sciences, Zhejiang Normal University, Jinhua, Zhejiang China; 2 Exercise and Metabolism Research Center, Zhejiang Normal University, Jinhua, Zhejiang China; 3 Medical Molecular Biology Laboratory, School of Medicine, Jinhua Polytechnic, Jinhua, China; 4 School of Sports Science and Physical Education, Research Center of Sports and Health Science, Northeast Normal University, 5268 Renmin Street, Changchun, Jilin, 130024 China; 5 Department of Colorectal Anal Surgery, Jinhua People’s Hospital, 267 Danxi East Road, Jinhua, 321007 Zhejiang China

**Correction: Diabetology & Metabolic Syndrome (2023) 15:154** **https://doi.org/10.1186/s13098-023-01130-w**

Following publication of the original article [1], the authors identified an error in affiliation and order. The revised affiliations and order are corrected in this erratum.
