# Supplementary material for: Exercise mitigates Dapagliflozin-induced skeletal muscle atrophy in STZ-induced diabetic rats
Source: Diabetol Metab Syndr. 2023 Jul 12;15:154. doi: 10.1186/s13098-023-01130-w (PMC10337193; doi:10.1186/s13098-023-01130-w)

# Original figures of experimental data

Note: This file contains part of the experimental data, and the author will provide the complete data unconditionally upon reasonable request.

**Figure 2**

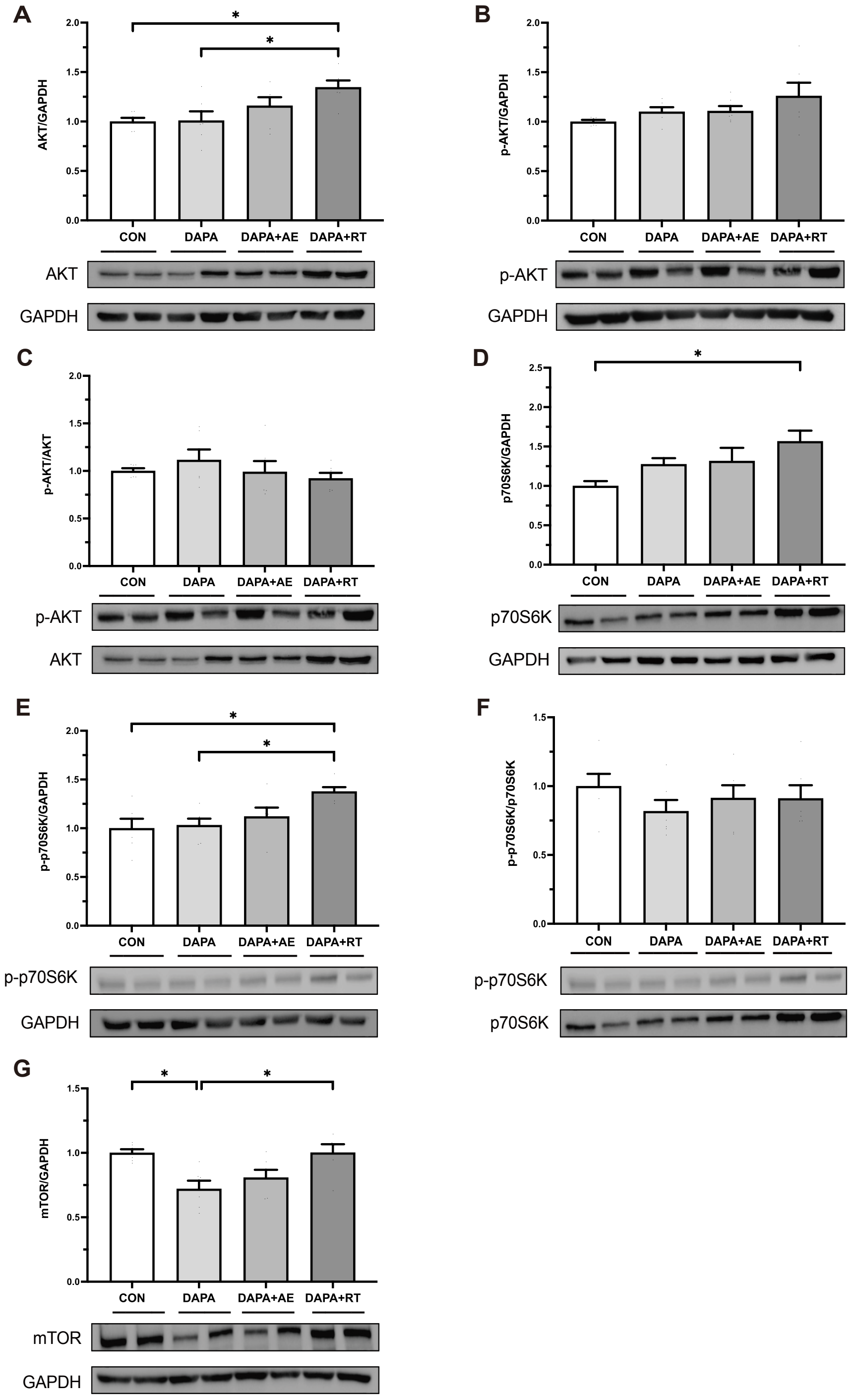


**Figure 3**

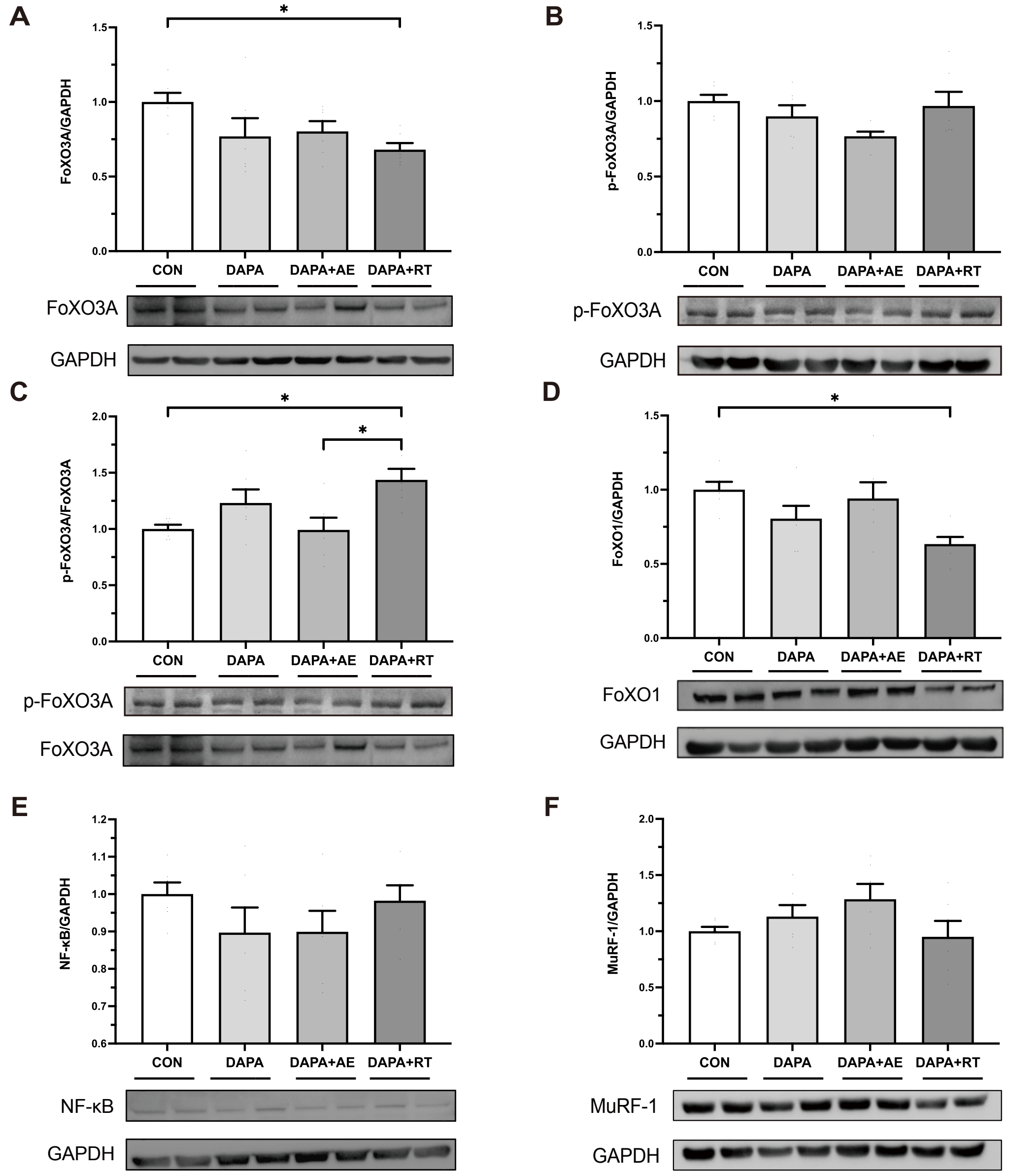

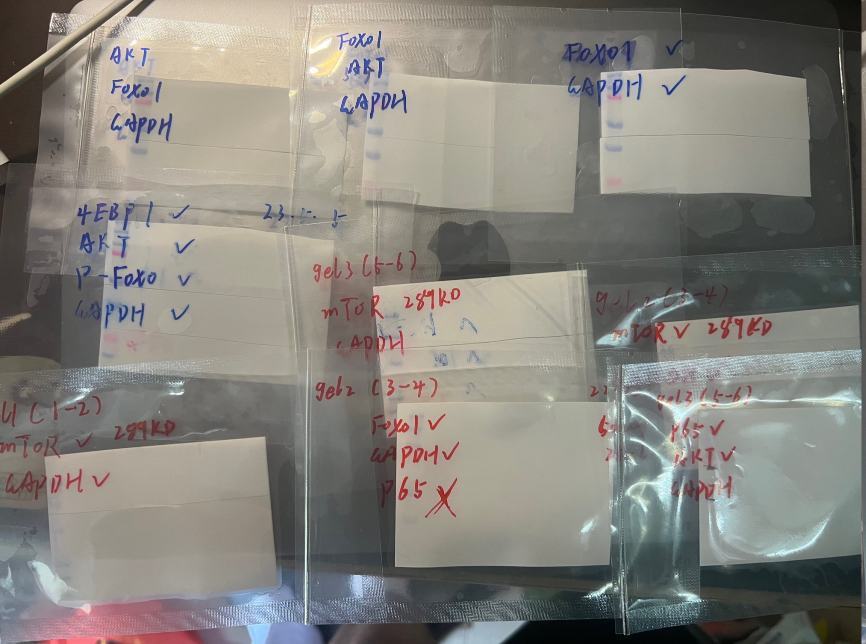
AKT
FoxO1
GAPDH
FoxO1 ✓
GAPDH ✓
4EBP1 ✓
AKT ✓
P-FoxO ✓
GAPDH ✓
gel3 (5-6)
mTOR 289KD
gel2 (3-4)
FoxO1 ✓
GAPDH ✓
P65 ✗
mTOR ✓ 289KD
GAPDH ✓
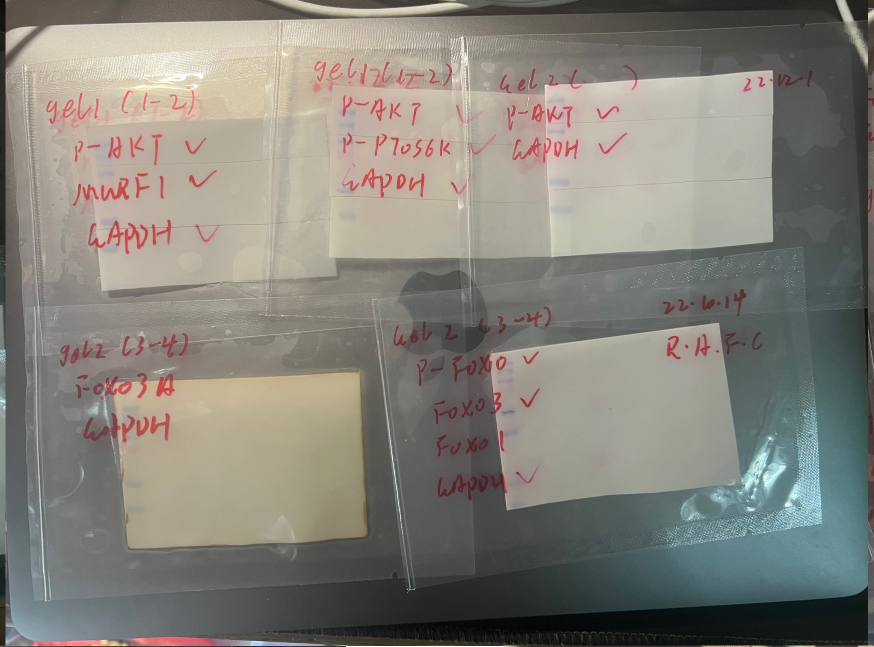
gel1 (1-2)
P-AKT ✓
MuRF1 ✓
GAPDH ✓
P-AKT
P-P70S6K
GAPDH
22.12.1
gel2 (3-4)
FoxO3A
GAPDH
gel2 (3-4)
P-FoxO ✓
FoxO3 ✓
FoxO1
GAPDH ✓
22.6.14
R.A.F.C
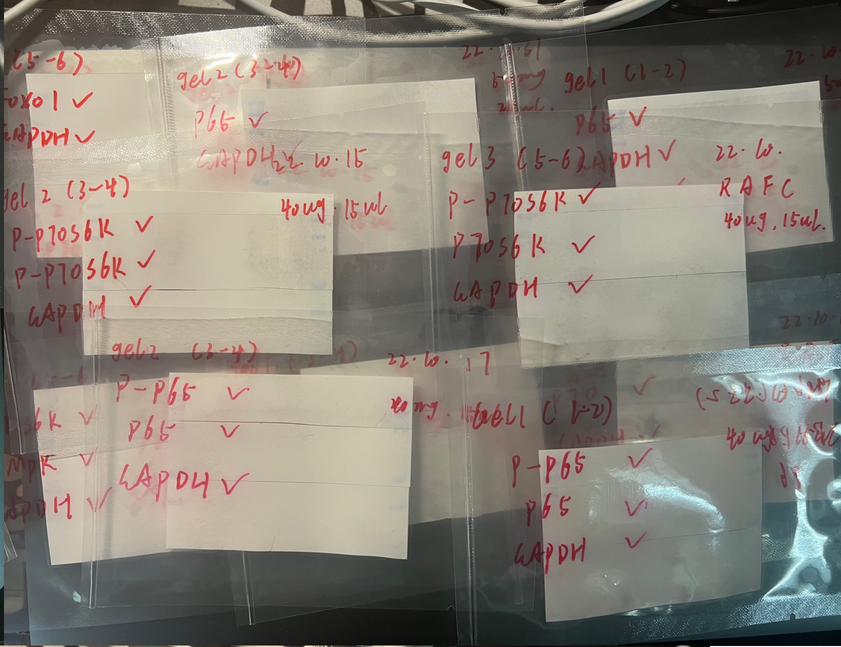
gel2 (3-4)
P65 ✓
gel1 (1-2)
gel3 (5-6)
gel 2 (3-4)
P-P70S6K ✓
P70S6K ✓
GAPDH ✓
RAFC
P-P65 ✓
P65 ✓
GAPDH ✓
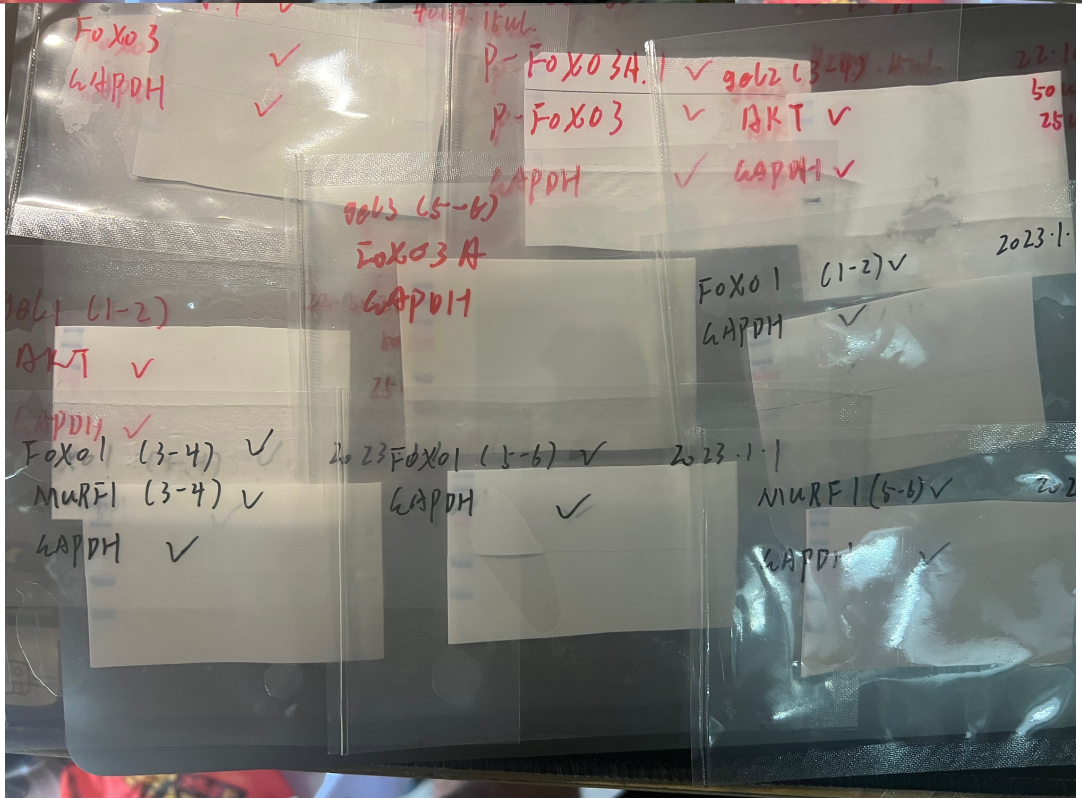
FoxO3
GAPDH
P-FoxO3A ✓
P-FoxO3 ✓
GAPDH
AKT ✓
gel3 (5-6)
FoxO3A
GAPDH
2023.1.
gel1 (1-2)
AKT ✓
FoxO1 (1-2) ✓
FoxO1 (3-4) ✓
MuRF1 (3-4) ✓
GAPDH ✓
2023 FoxO1 (5-6) ✓
2023.1.1
MuRF1 (5-6) ✓
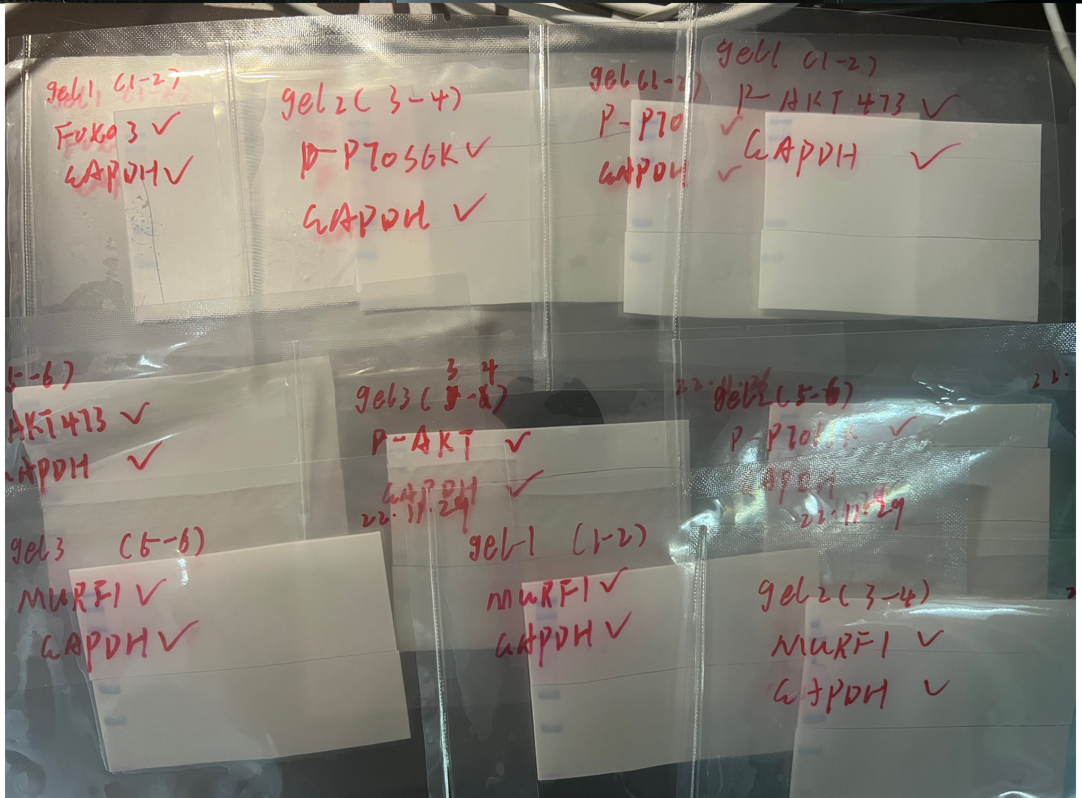
gel1 (1-2)
FoxO3 ✓
GAPDH ✓
gel2 (3-4)
P-P70S6K ✓
GAPDH ✓
P-AKT 473 ✓
GAPDH ✓
AKT473 ✓
GAPDH ✓
P-AKT ✓
gel3 (5-6)
MuRF1 ✓
GAPDH ✓
gel1 (1-2)
MuRF1 ✓
GAPDH ✓
gel2 (3-4)
MuRF1 ✓
GAPDH ✓

# Part 1. WB figures

# AKT

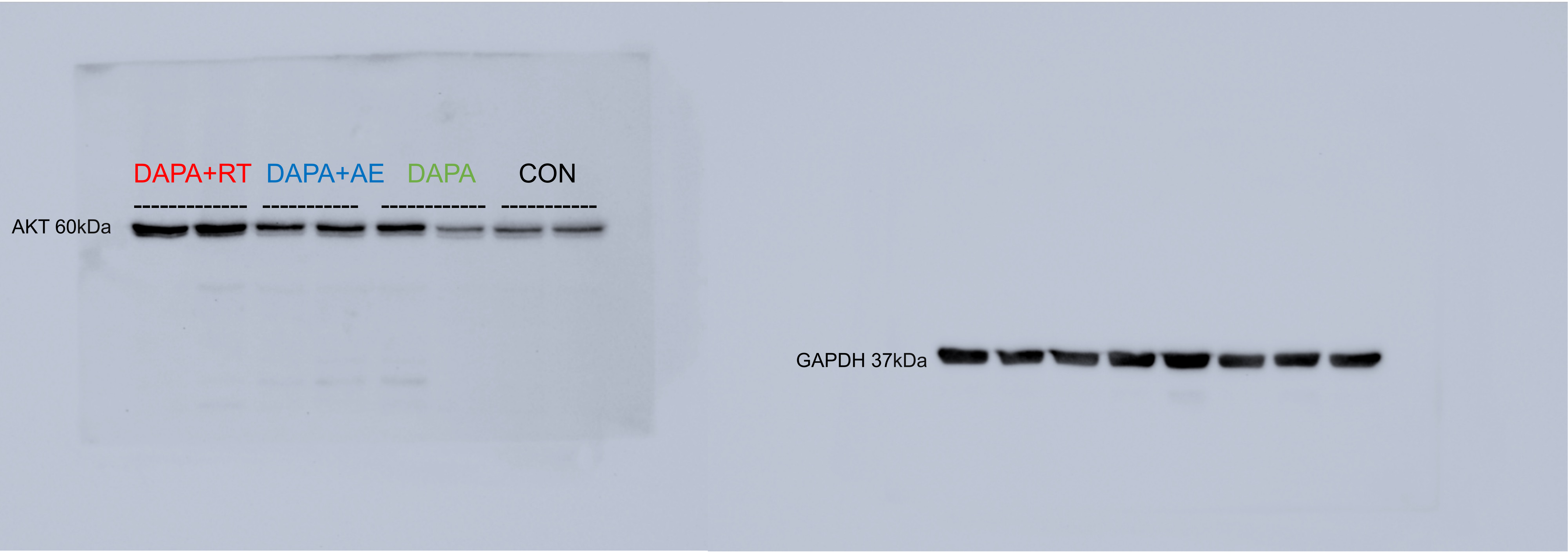


Note: Due to the high protein concentration, the exposure time is very short, resulting in no clear display of marker, but not lack of marker.

# AKT

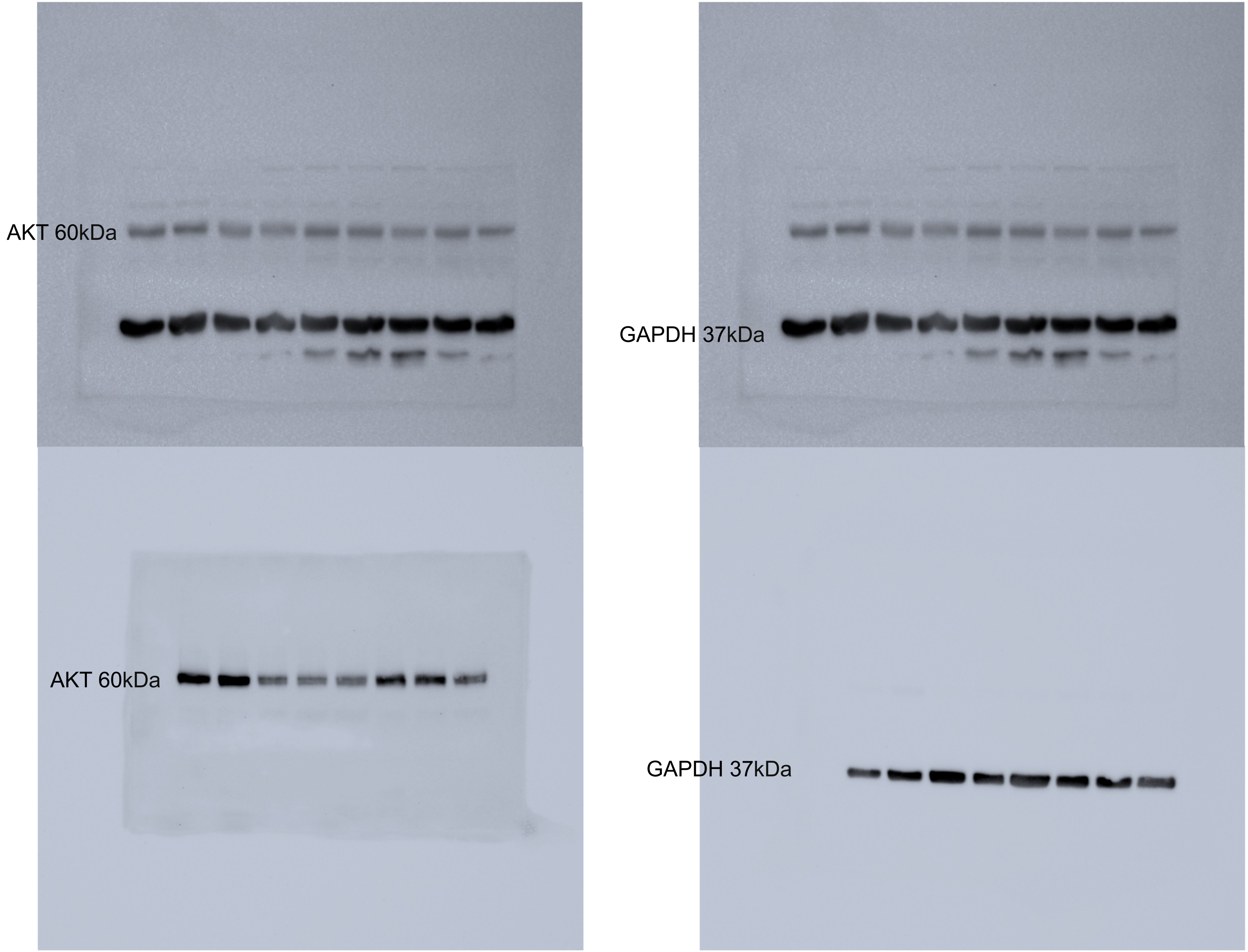

# NF-κB

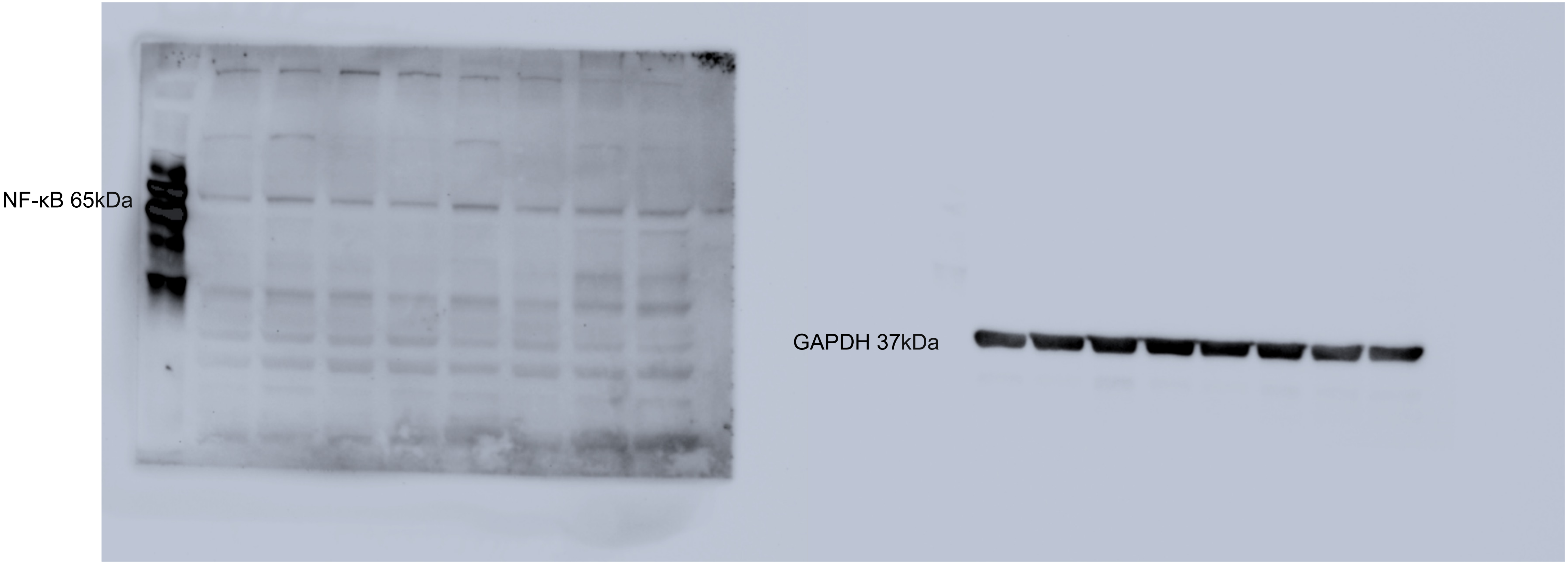

# NF-κB

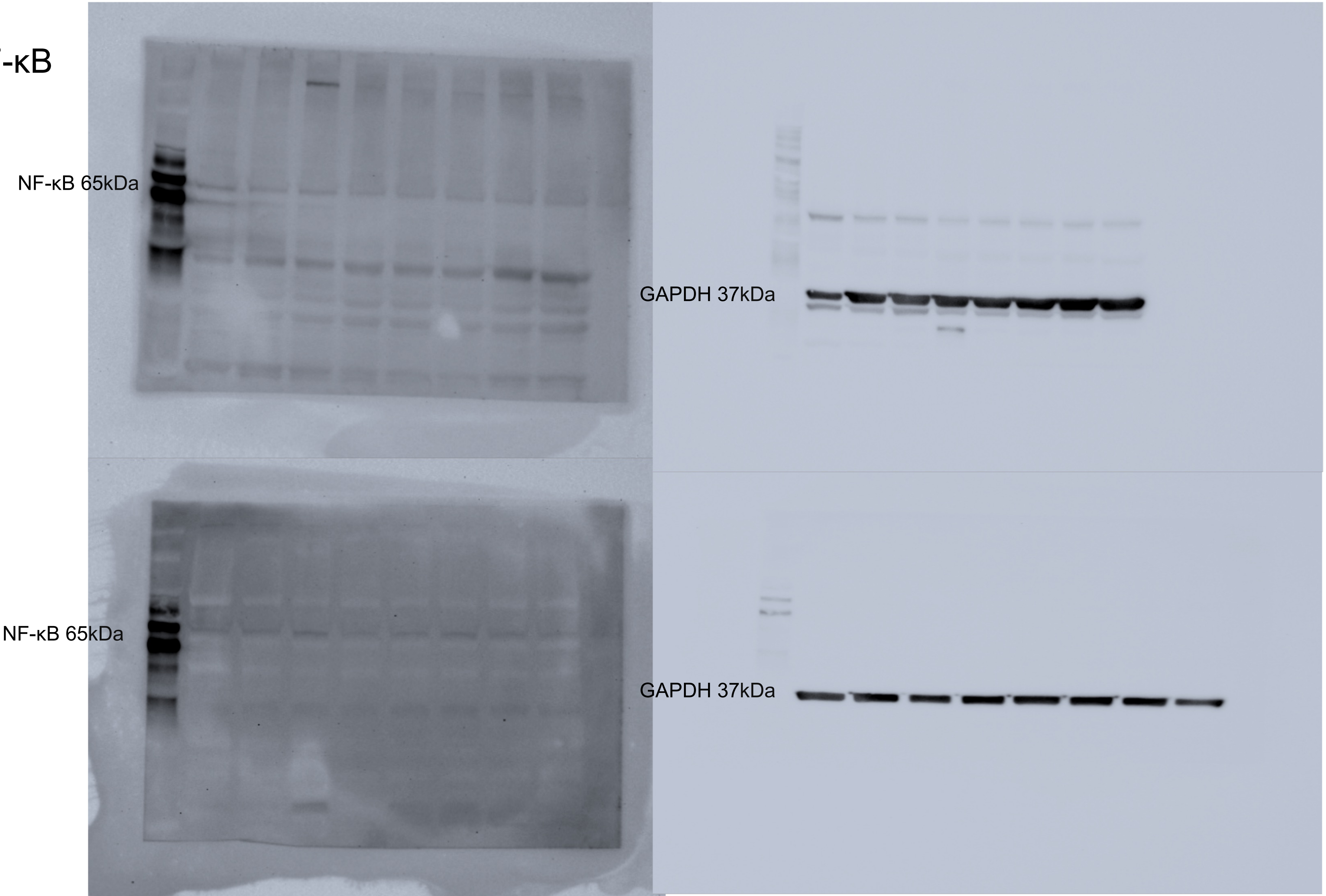

# P-AKT

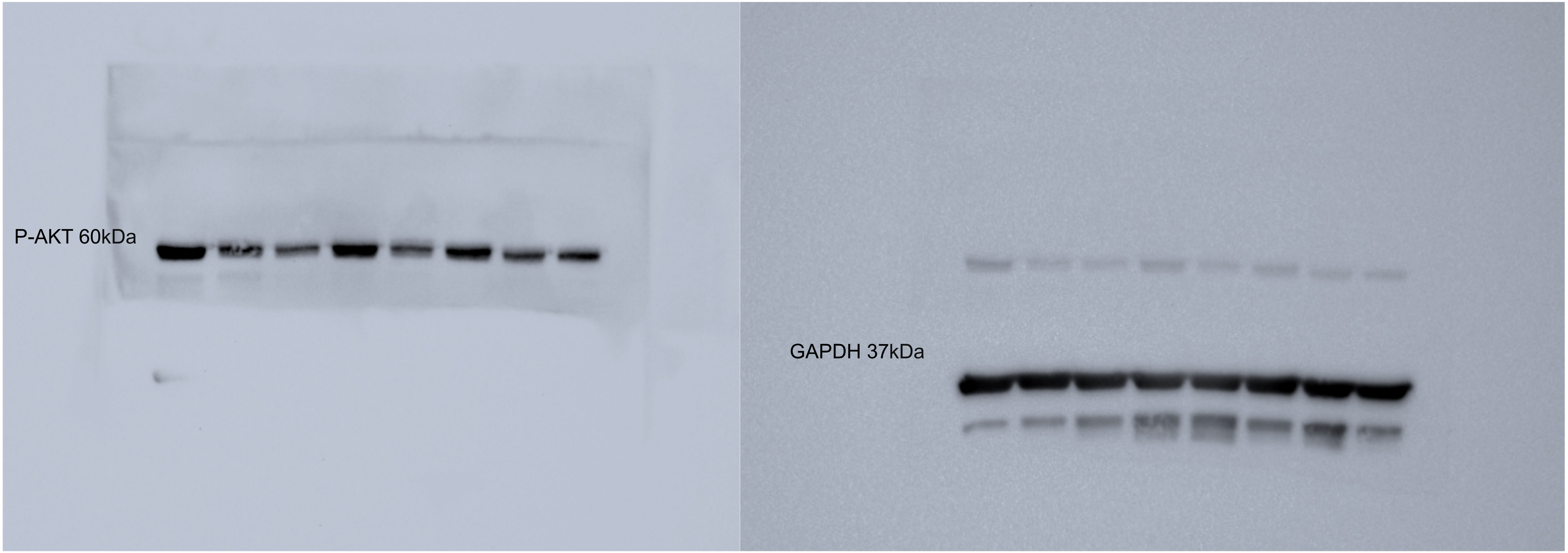


Note: Due to the high protein concentration, the exposure time is very short, resulting in no clear display of marker, but not lack of marker.

# P-AKT

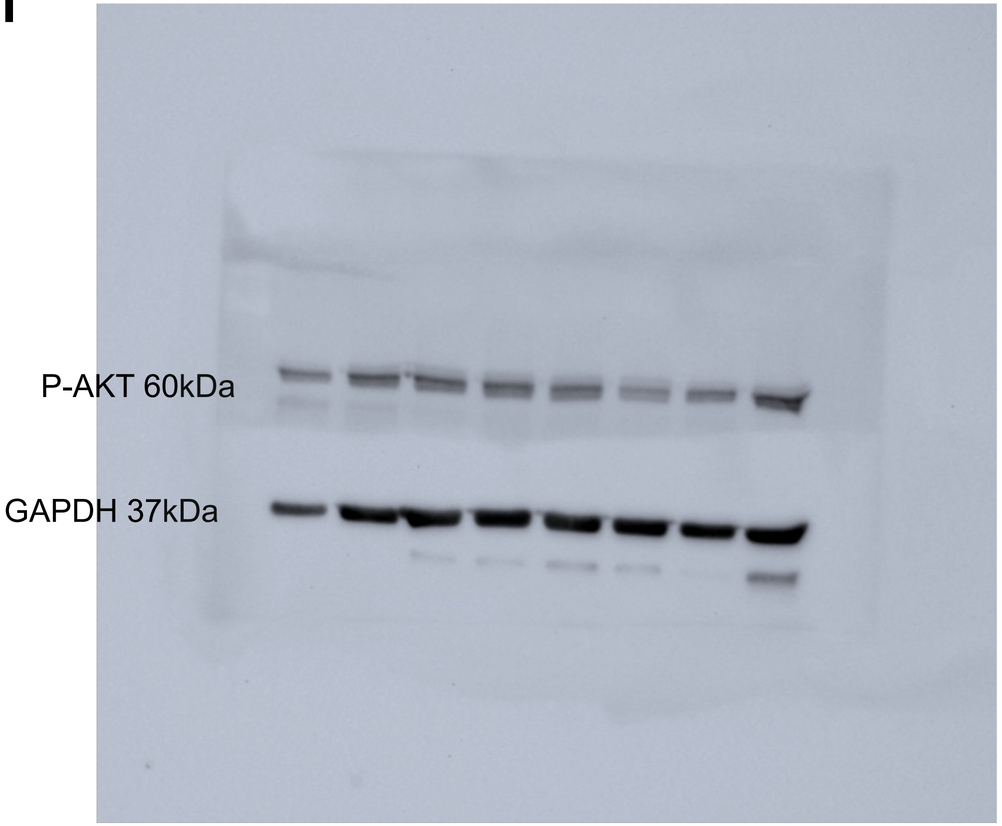


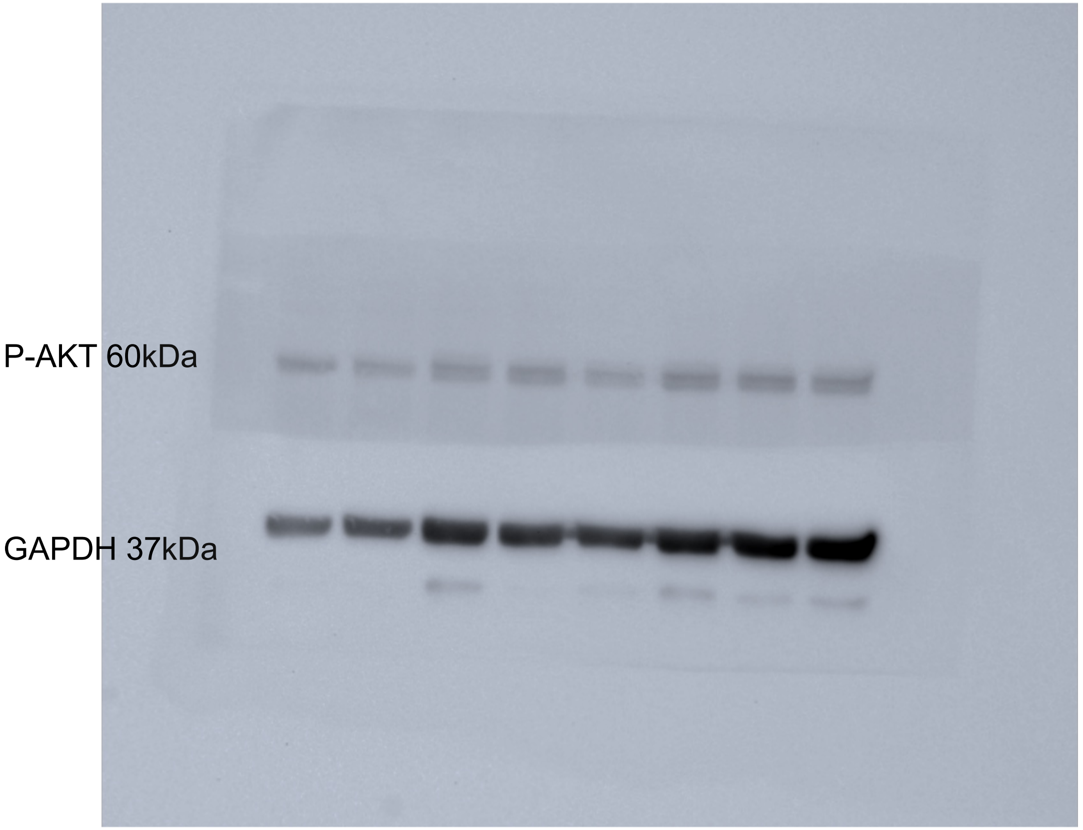

## p70S6K

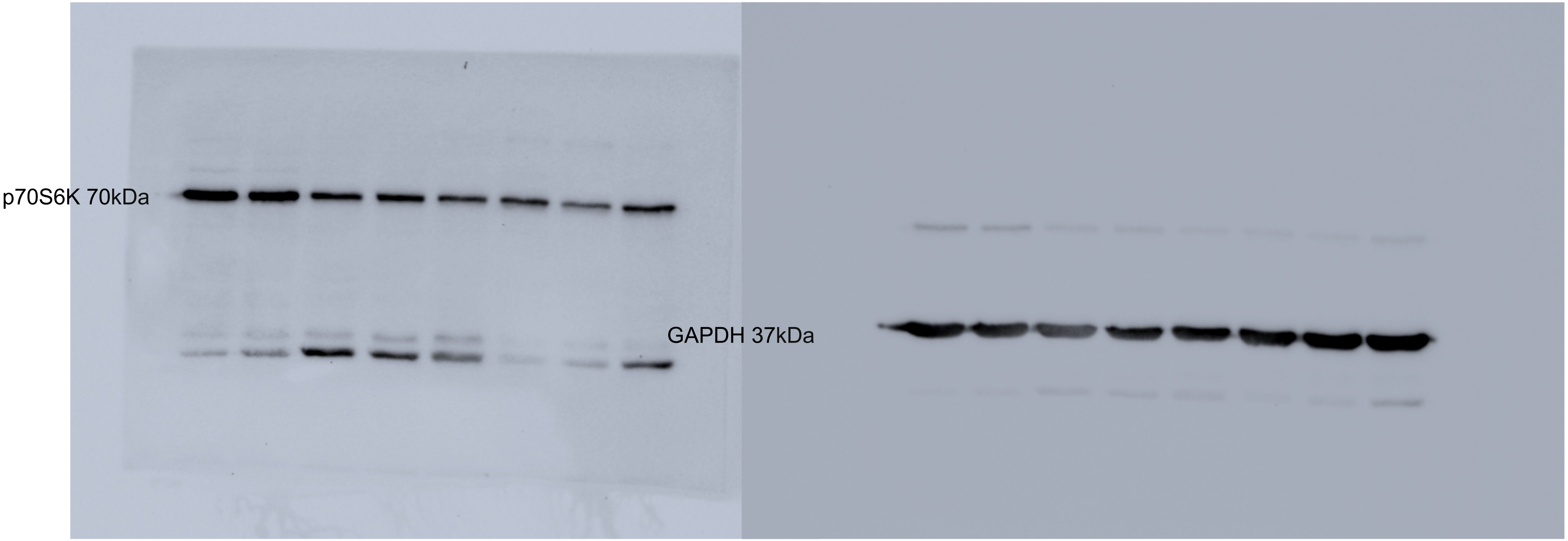


Note: Due to the high protein concentration, the exposure time is very short, resulting in no clear display of marker, but not lack of marker.

# p70S6K

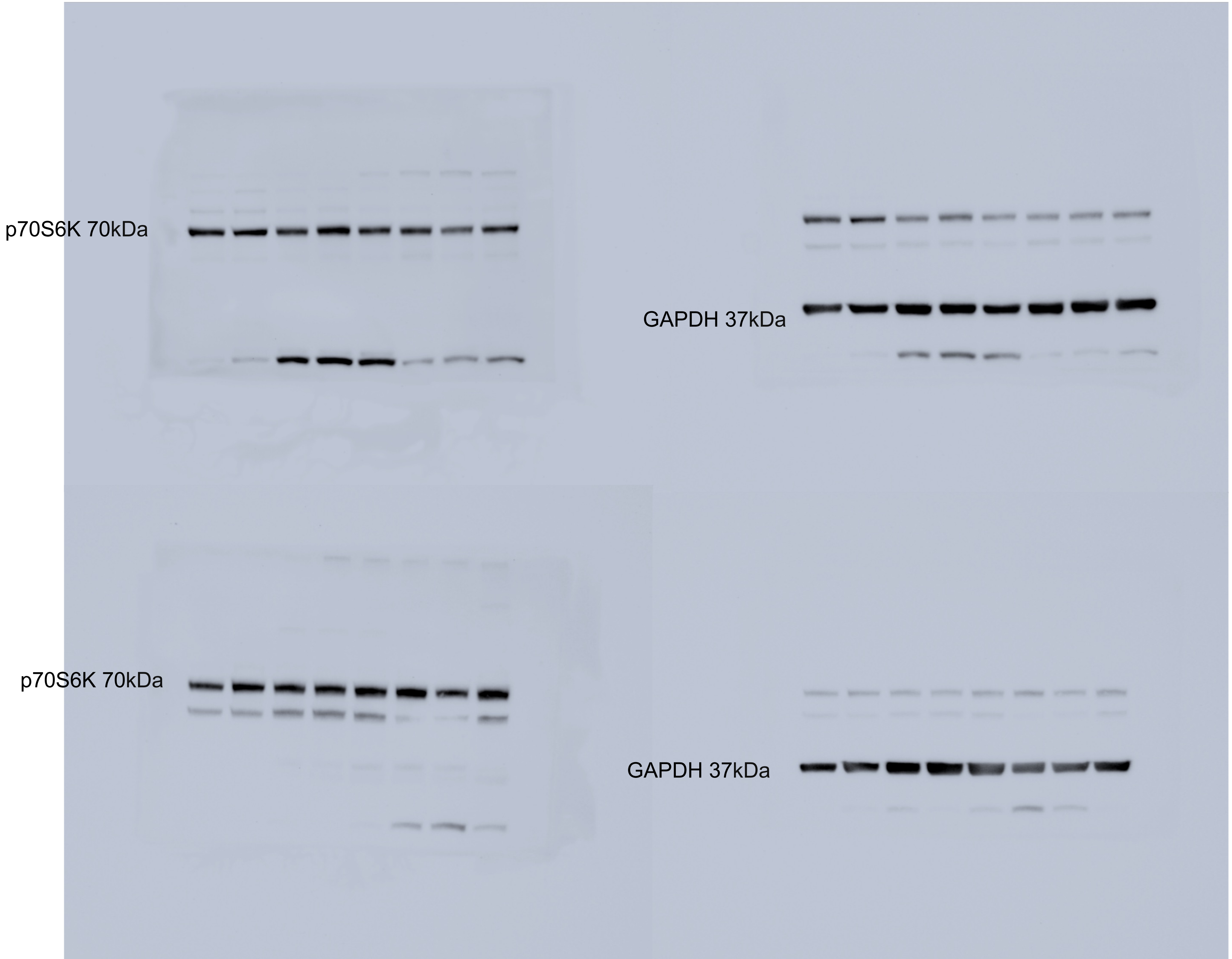

**p-p70S6K**

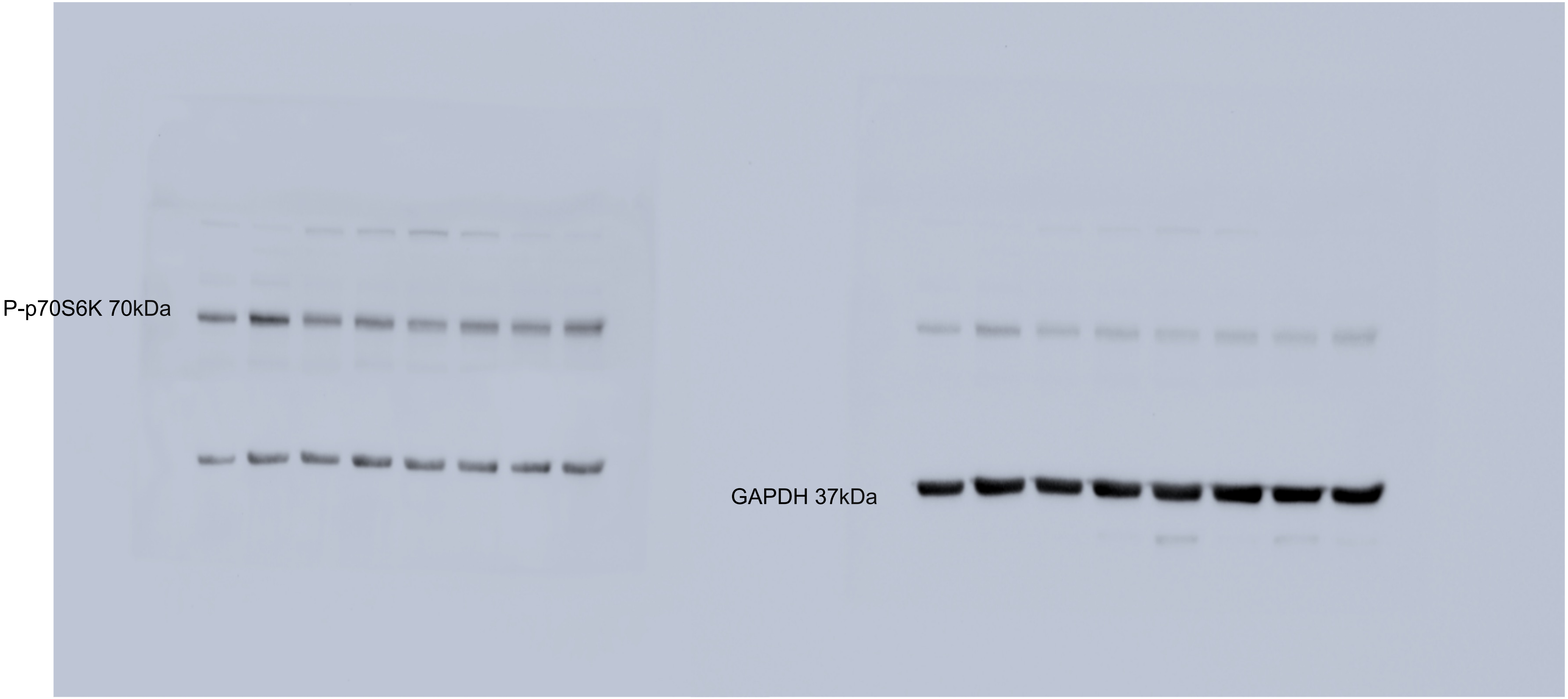


Note: Due to the high protein concentration, the exposure time is very short, resulting in no clear display of marker, but not lack of marker.

# p-p70S6K

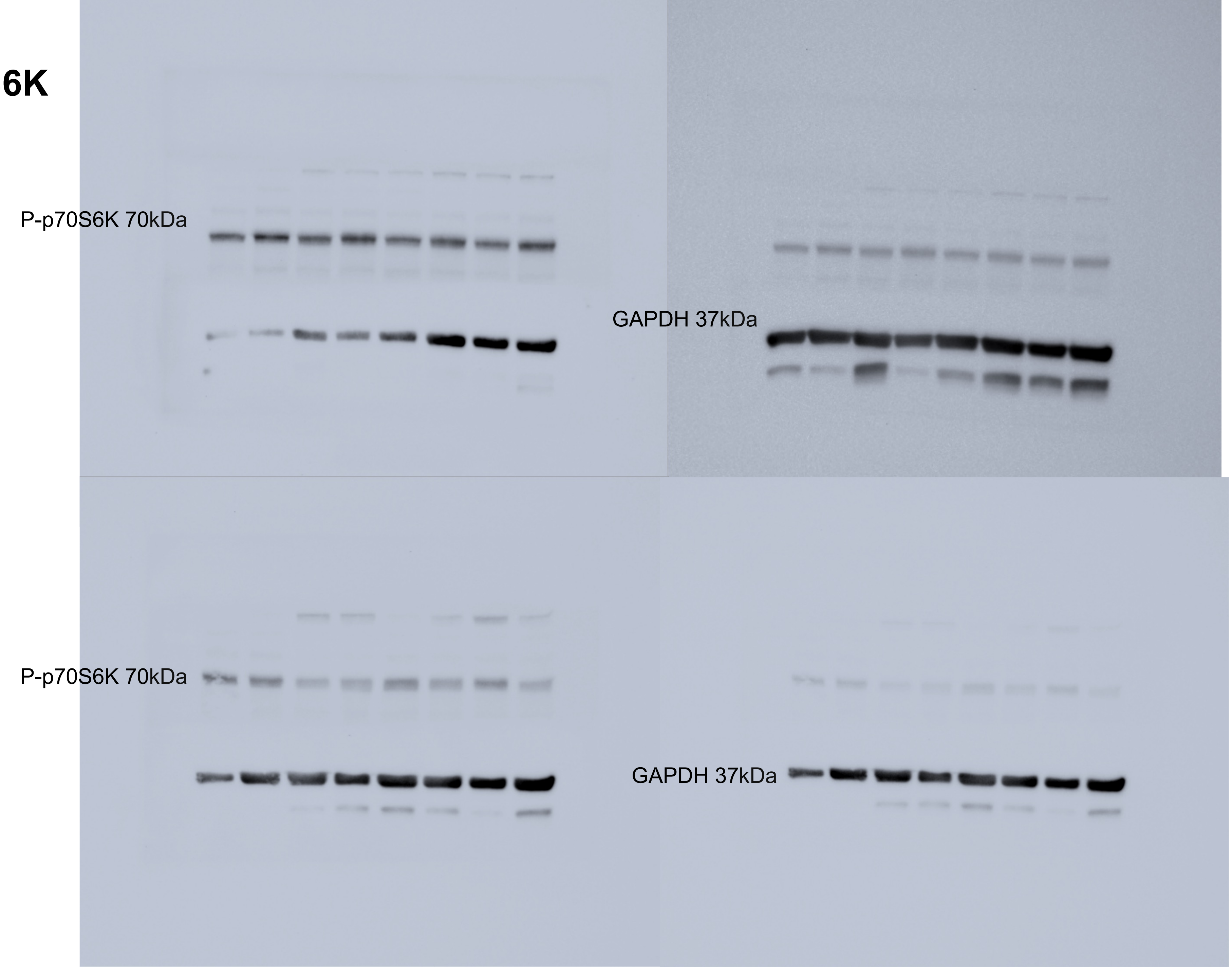

# P-FoXO3a

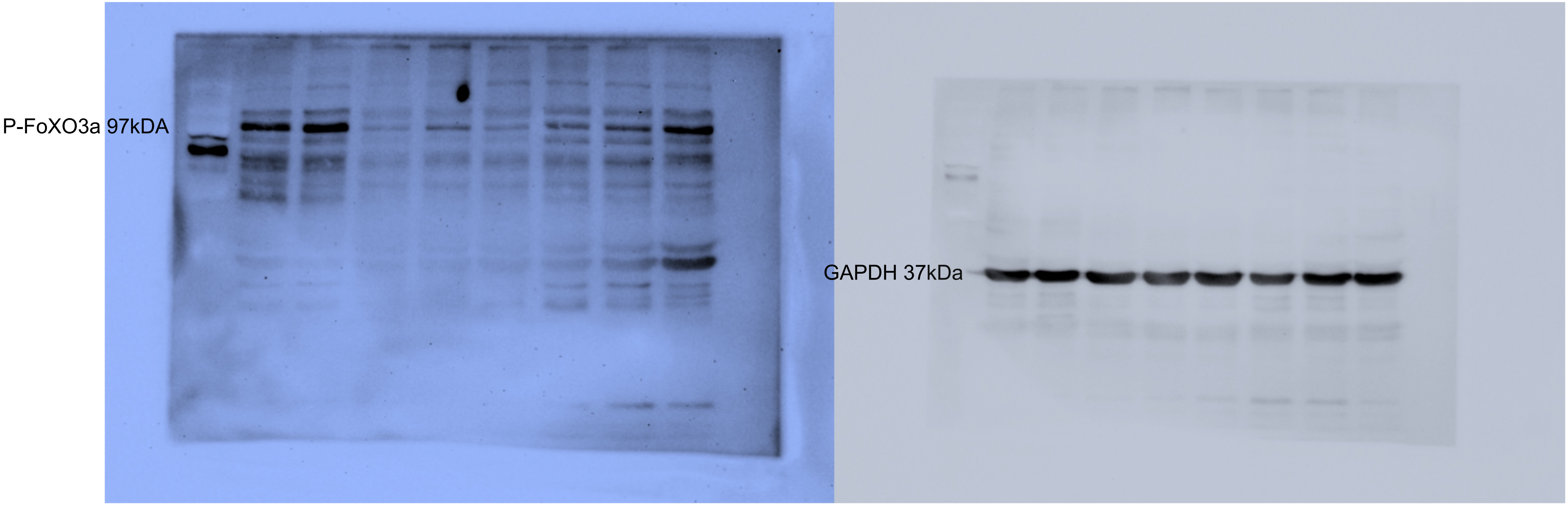


Note: Due to the high protein concentration, the exposure time is very short, resulting in no clear display of marker, but not lack of marker.

# P-FoXO3a

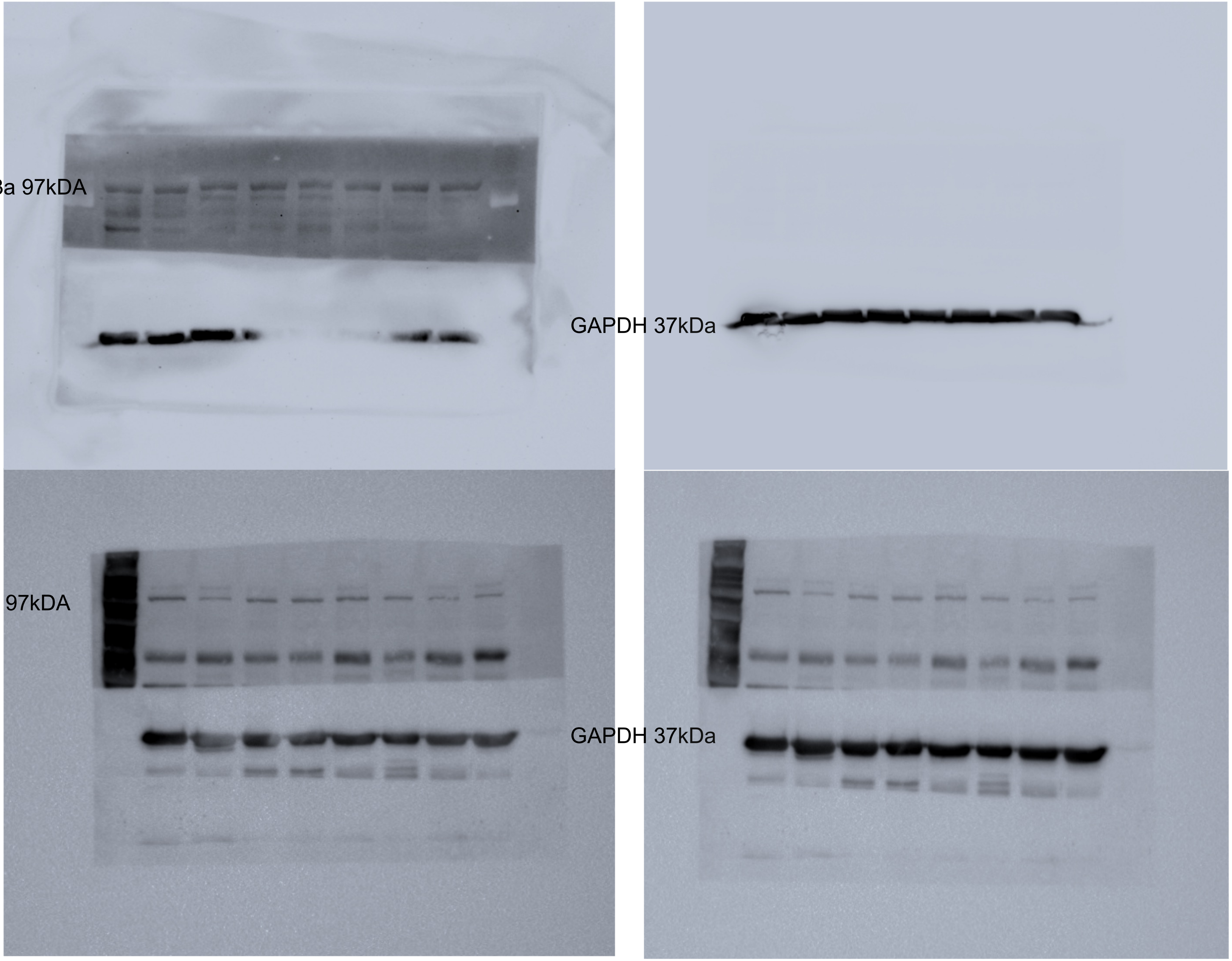

# FoXO1

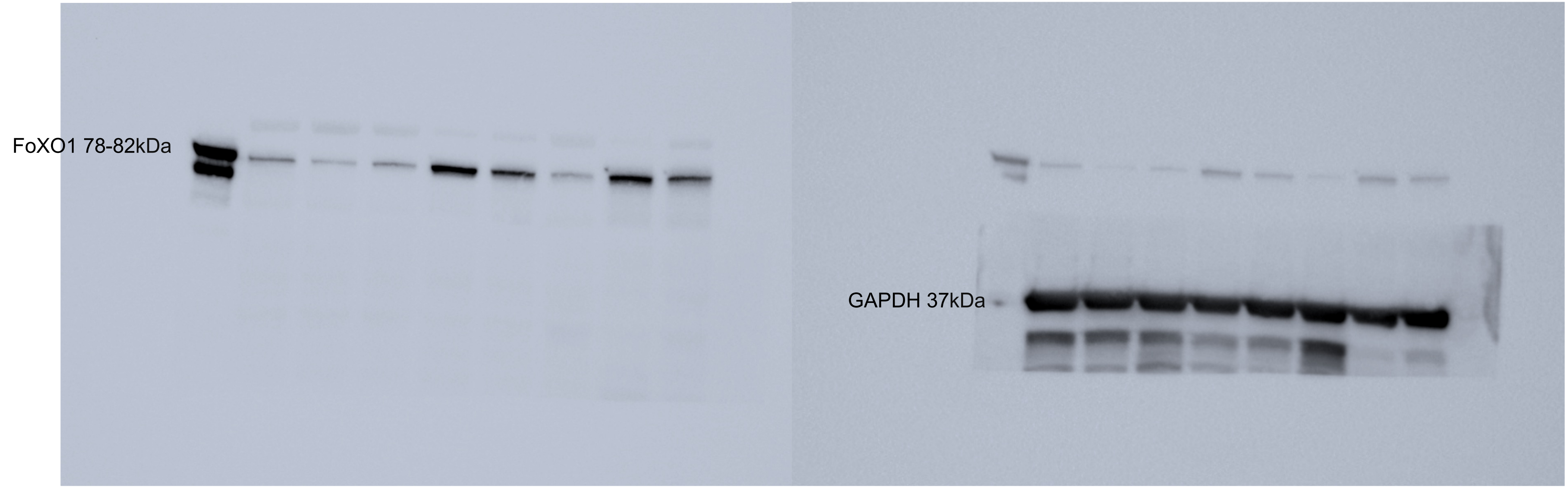


Note: Due to the high protein concentration, the exposure time is very short, resulting in no clear display of marker, but not lack of marker.

# FoXO1

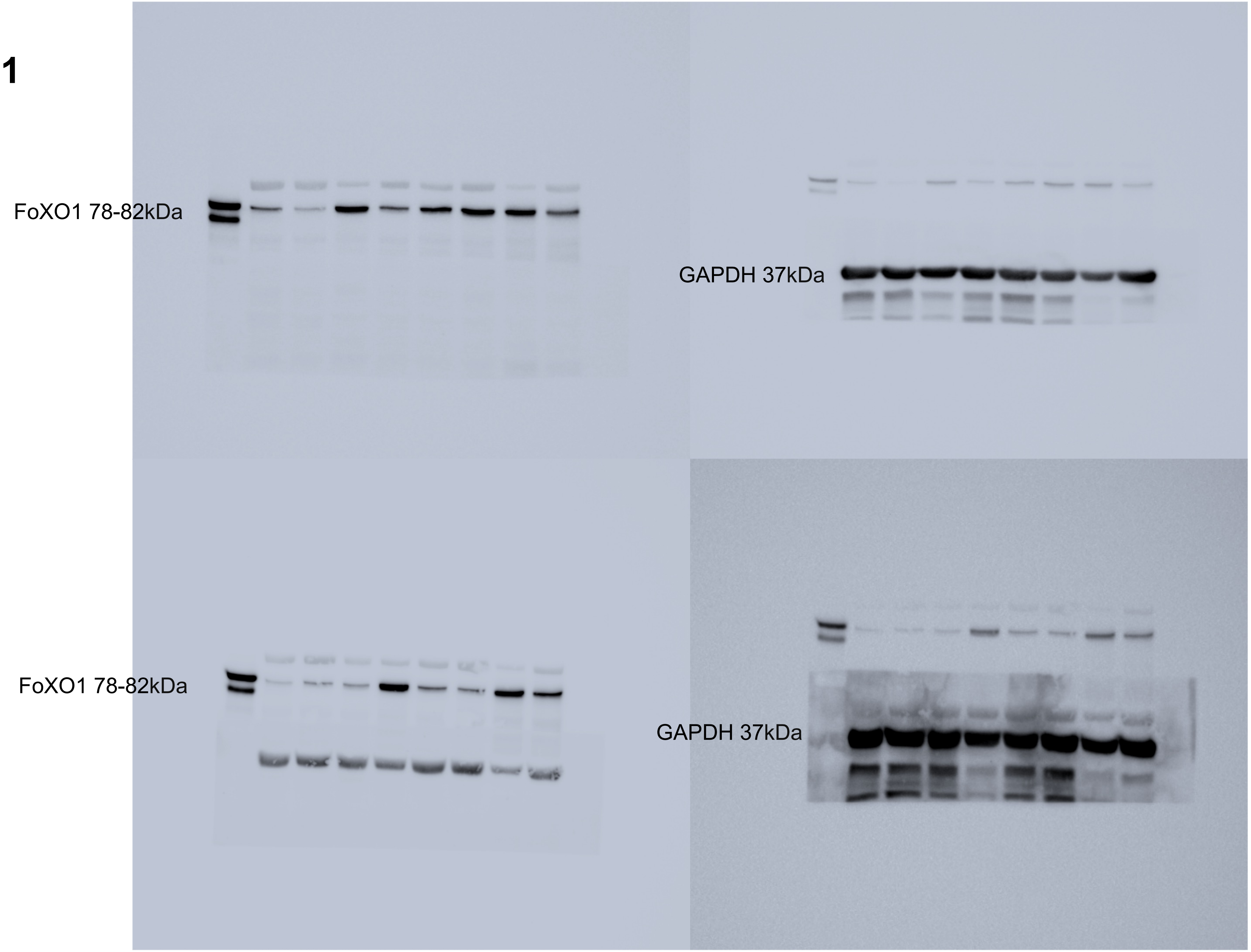

# FoXO3a

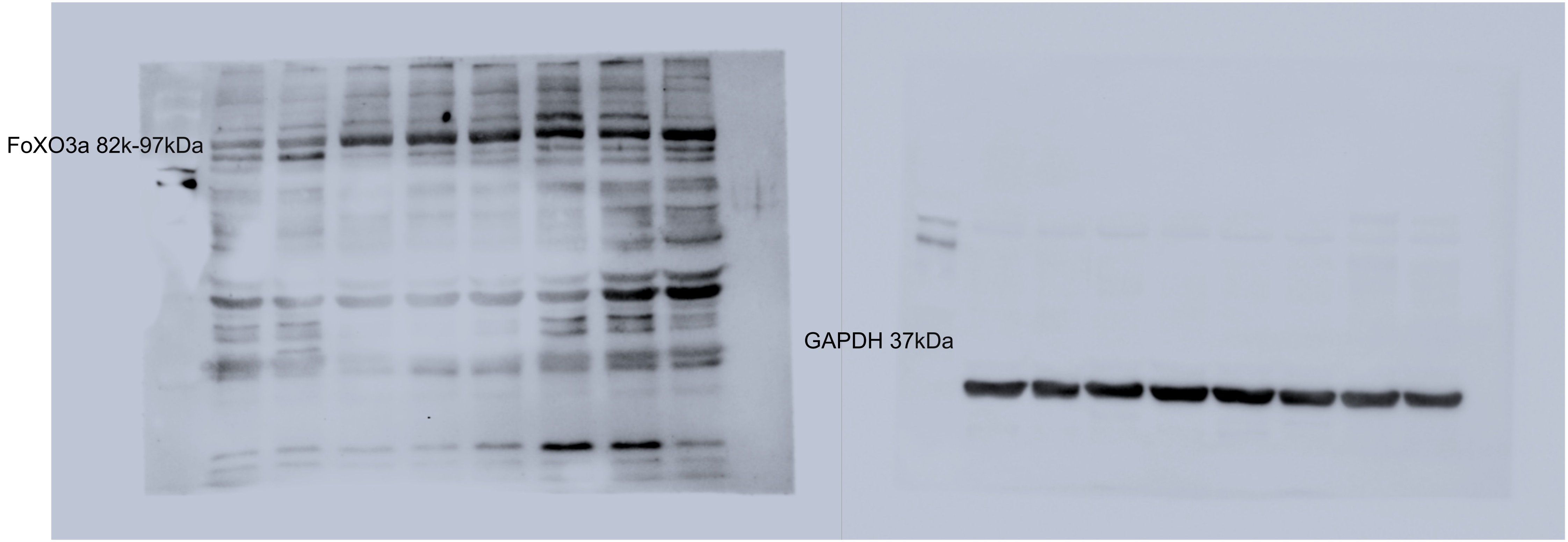

# FoXO3a

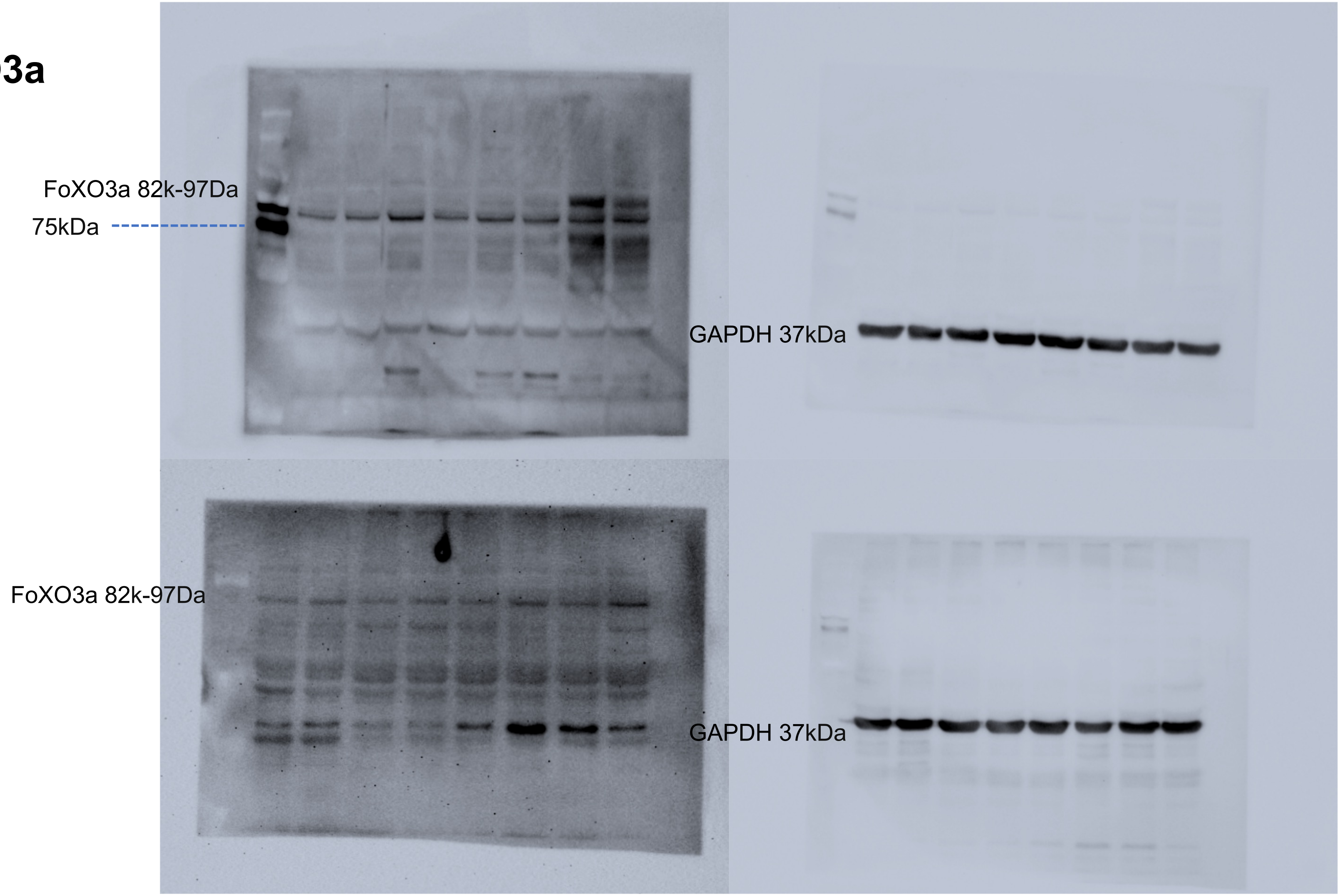
FoXO3a 82k-97Da
75kDa
GAPDH 37kDa
FoXO3a 82k-97Da
GAPDH 37kDa

**MuRF1**

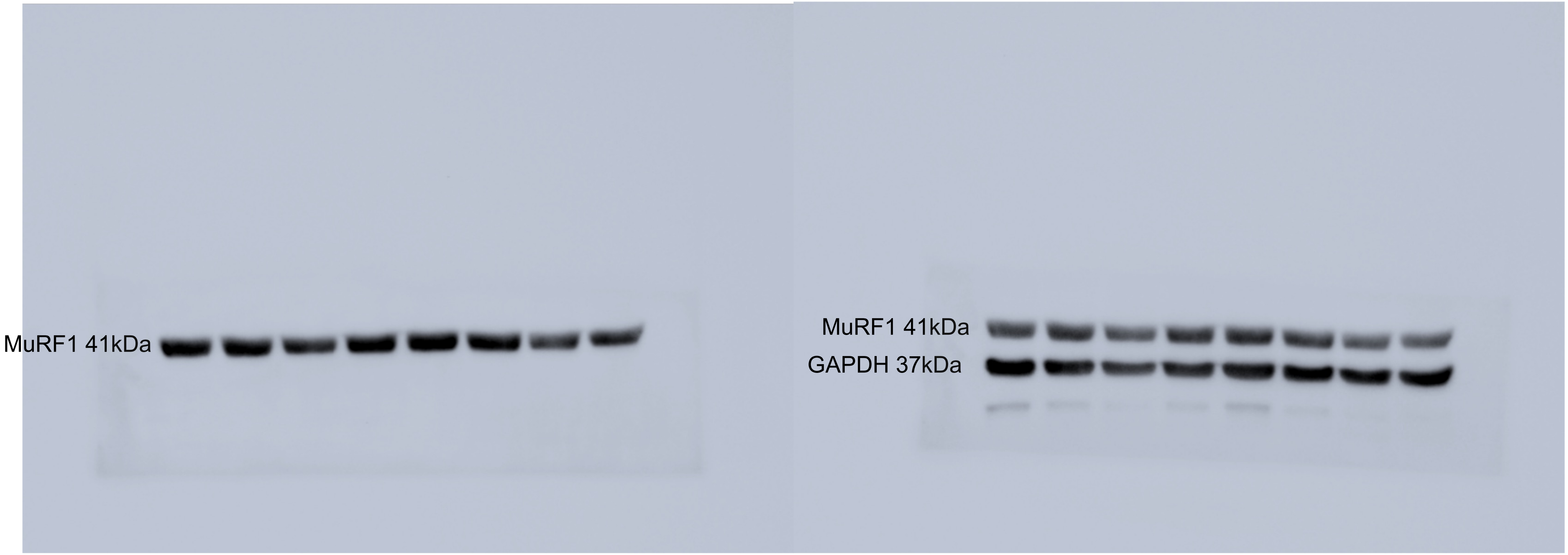


Note: Due to the high protein concentration, the exposure time is very short, resulting in no clear display of marker, but not lack of marker.

# MuRF1

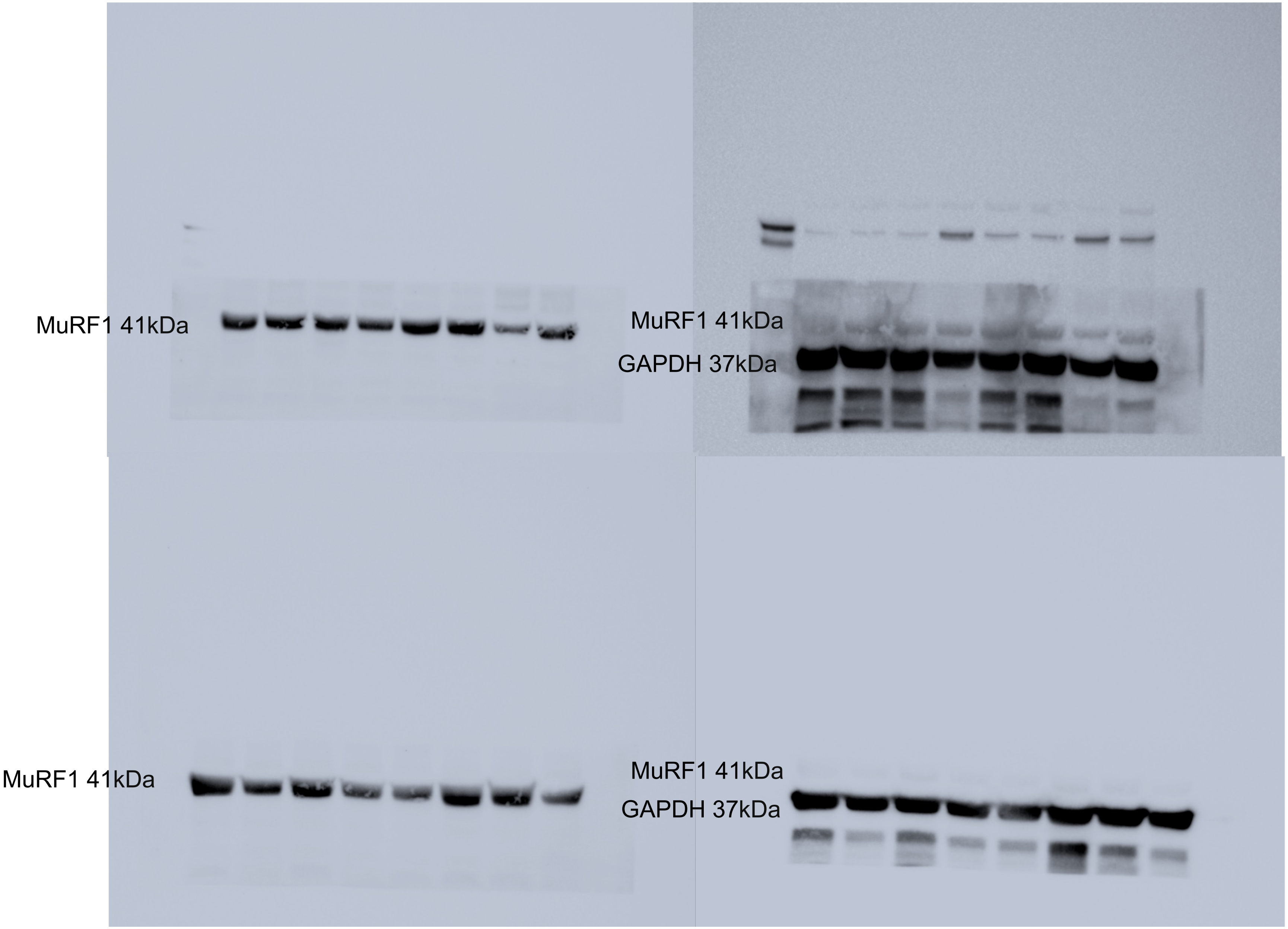

MuRF1 41kDa
MuRF1 41kDa
GAPDH 37kDa
MuRF1 41kDa
MuRF1 41kDa
GAPDH 37kDa

# mTOR

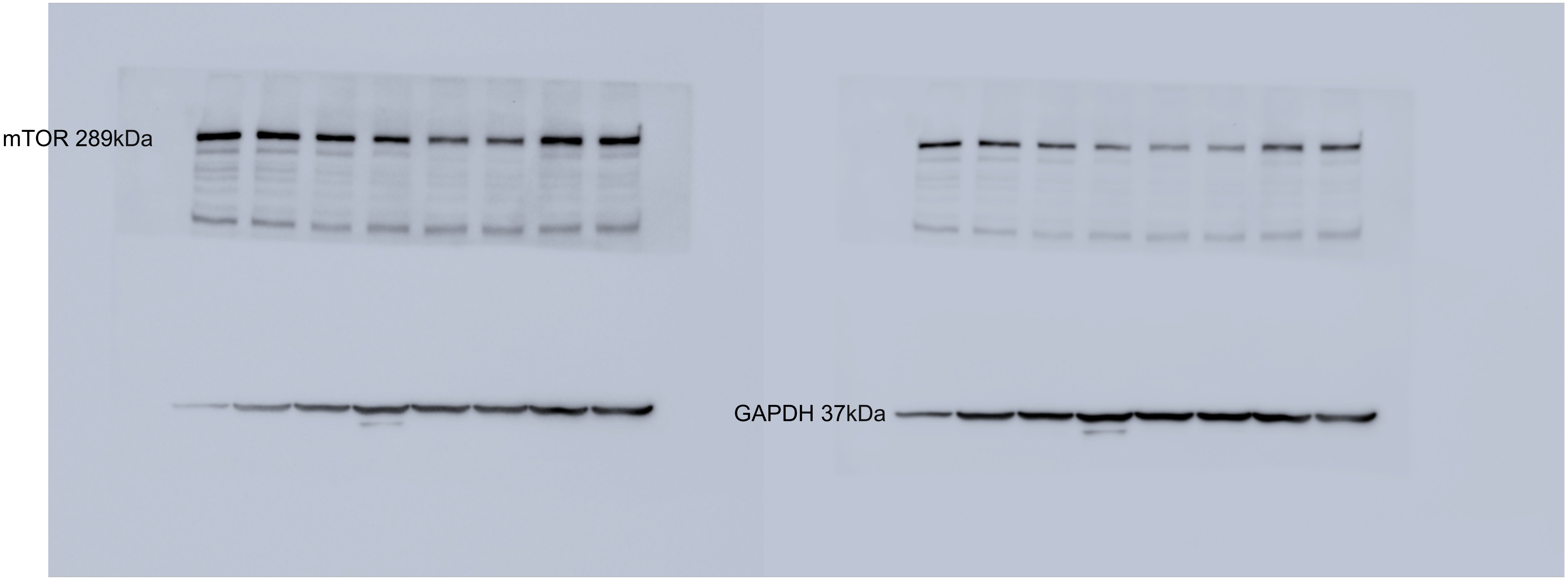


Note: This protein is electrophoreted with a layer of gel of different concentrations, with the upper layer concentration of 8% and the lower layer concentration of 12%.

# mTOR

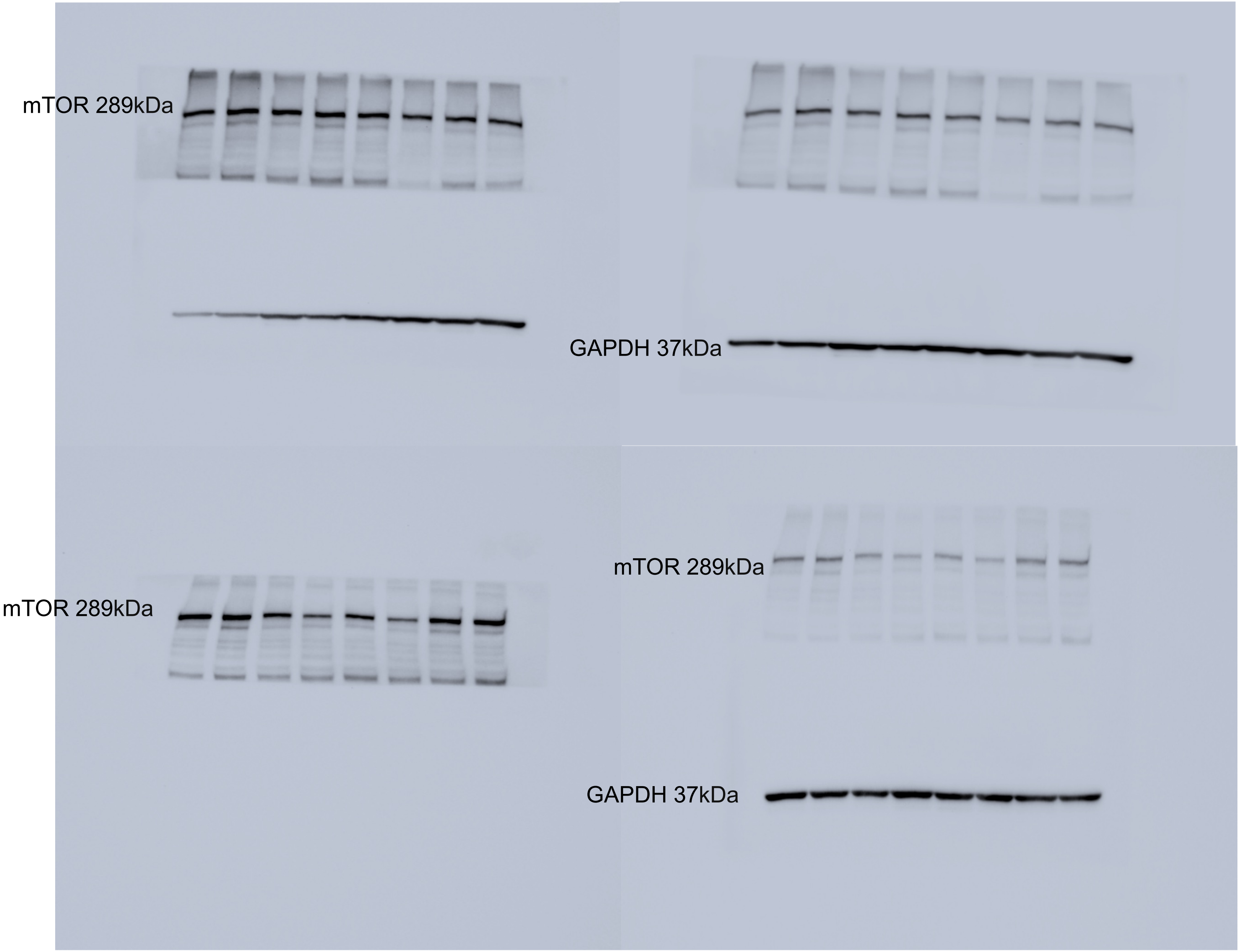
mTOR 289kDa
GAPDH 37kDa
mTOR 289kDa
mTOR 289kDa
GAPDH 37kDa

# Part 2. HE staining figures

DAPA+RT

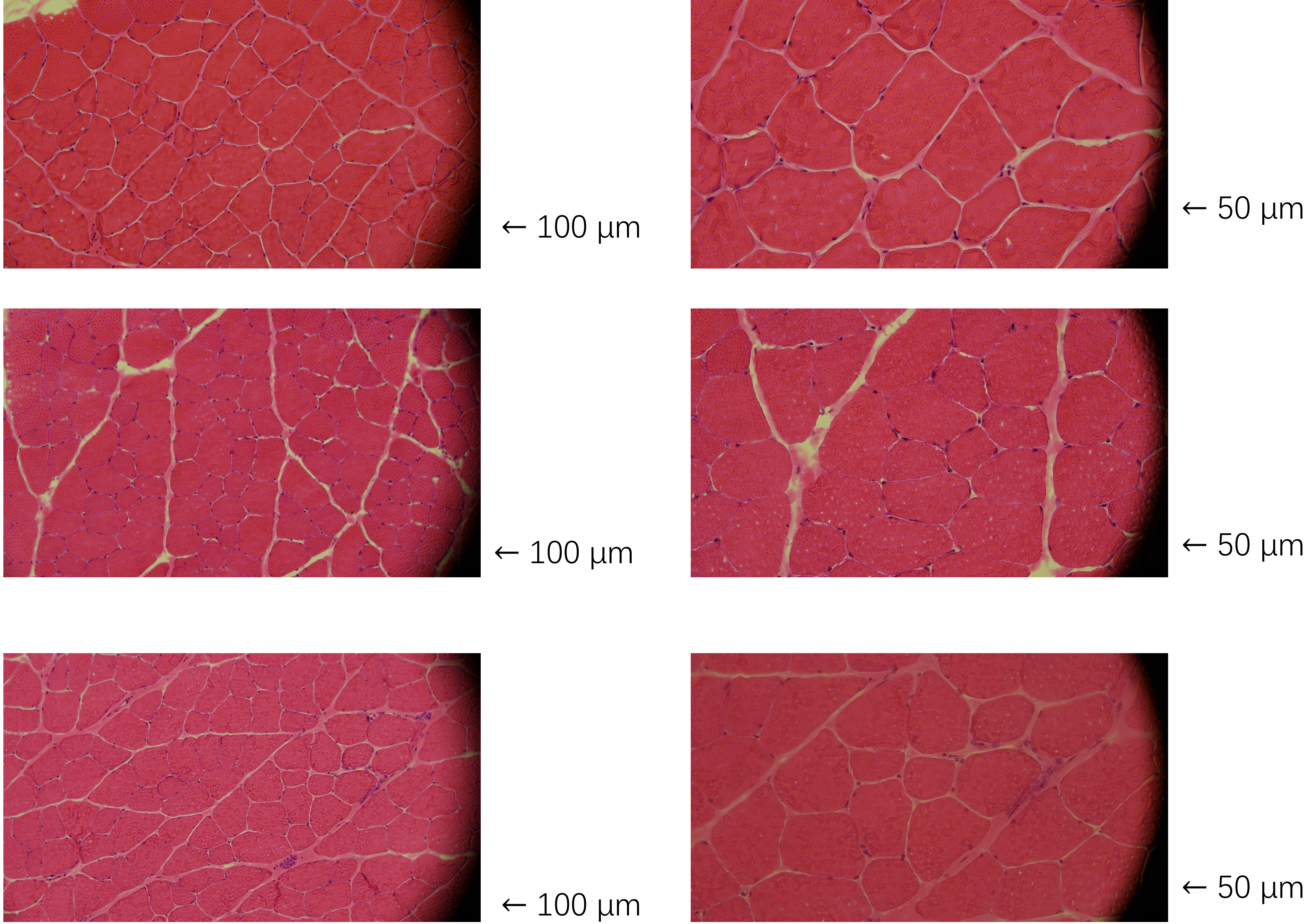

DAPA+AE

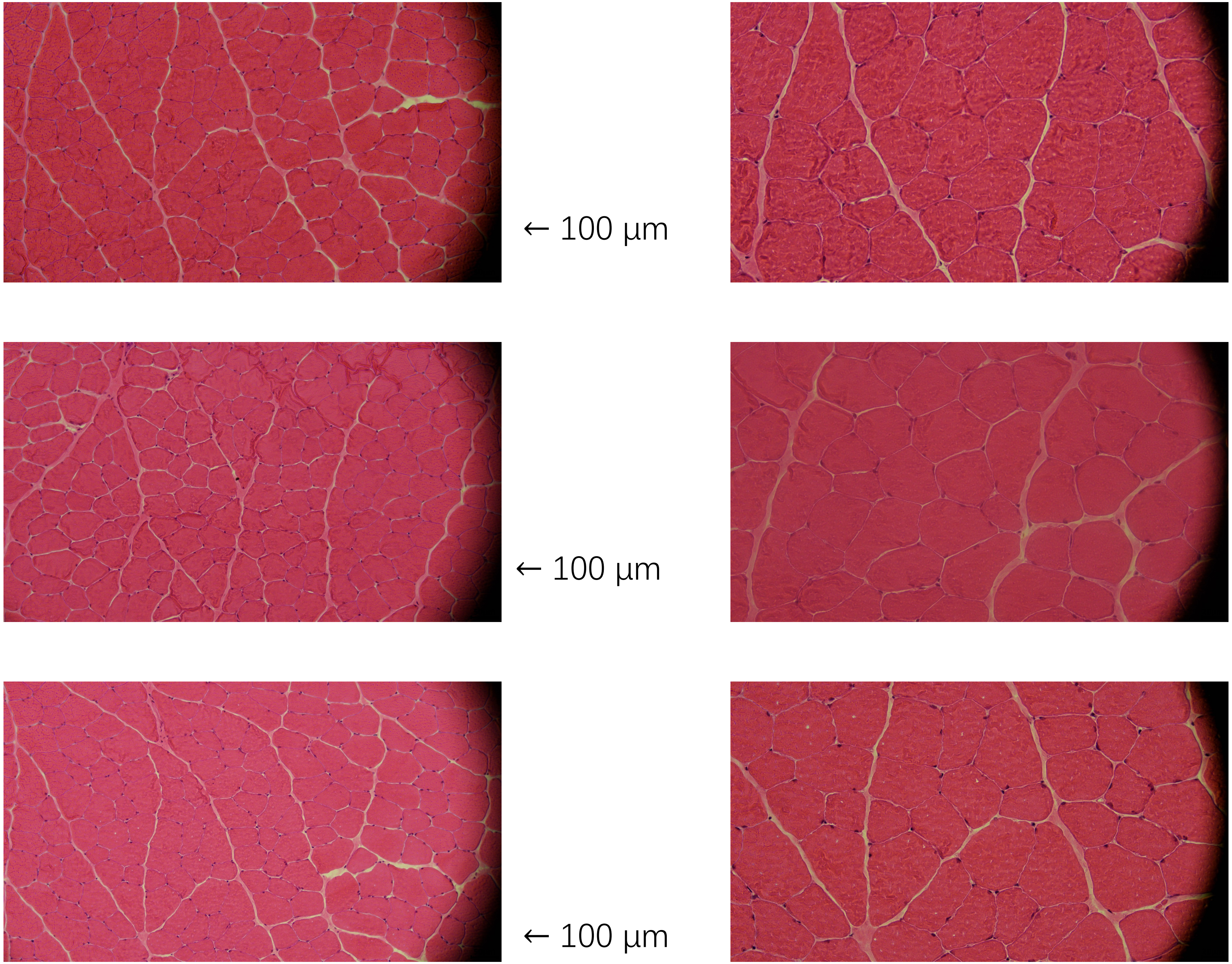

DAPA

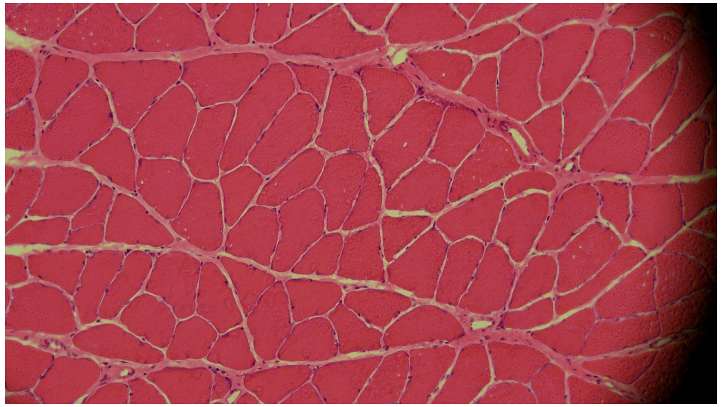

← 100 µm

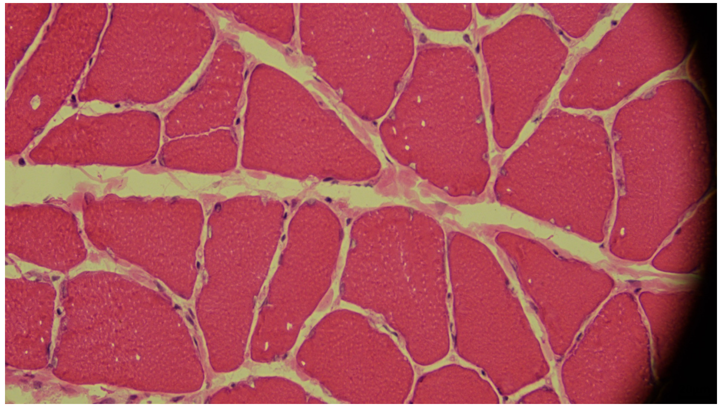

← 50 µm

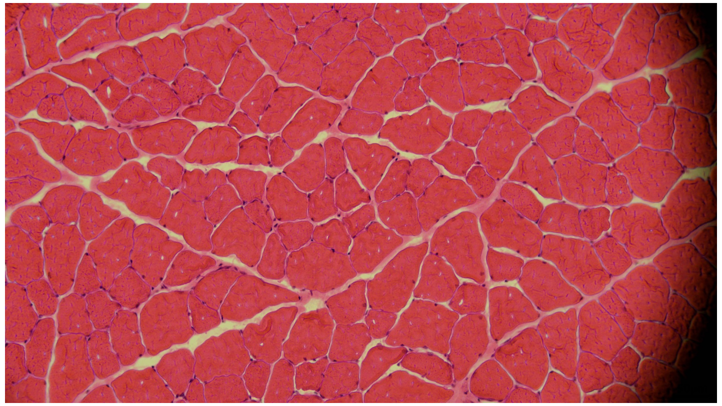

← 100 µm

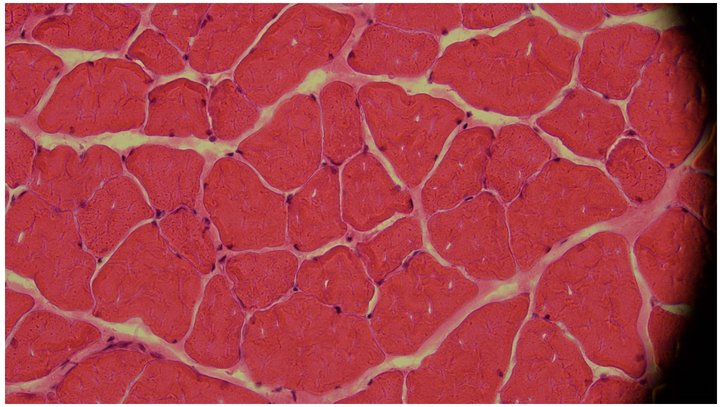

← 50 µm

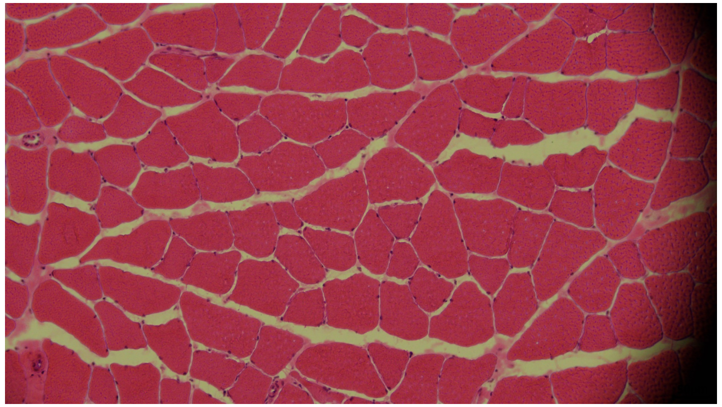

← 100 µm

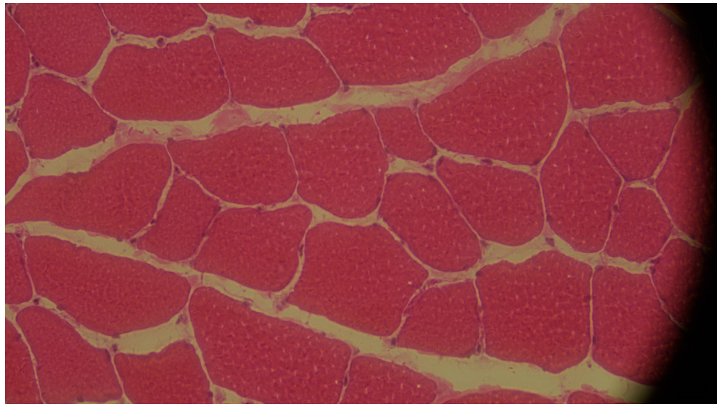

← 50 µm

CON

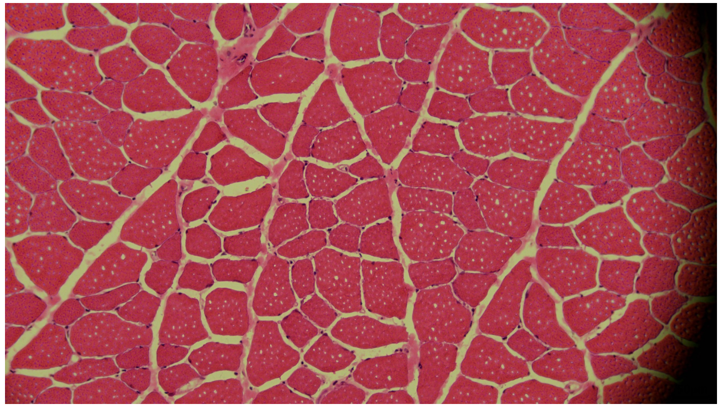

← 100 μm

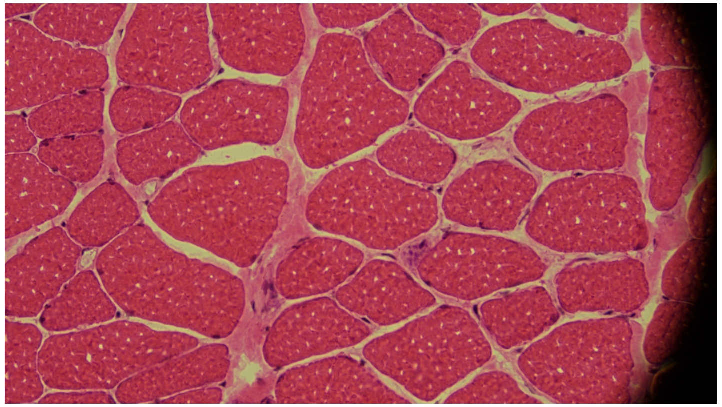

← 50 μm

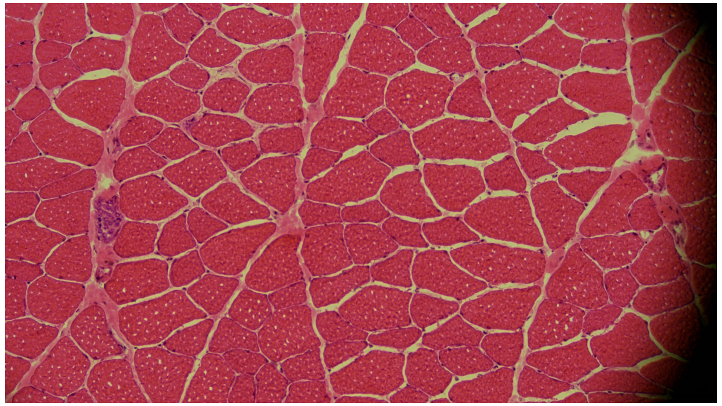

← 100 μm

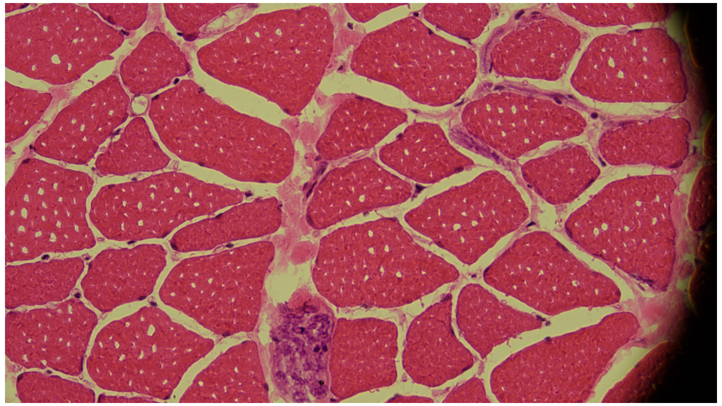

← 50 μm

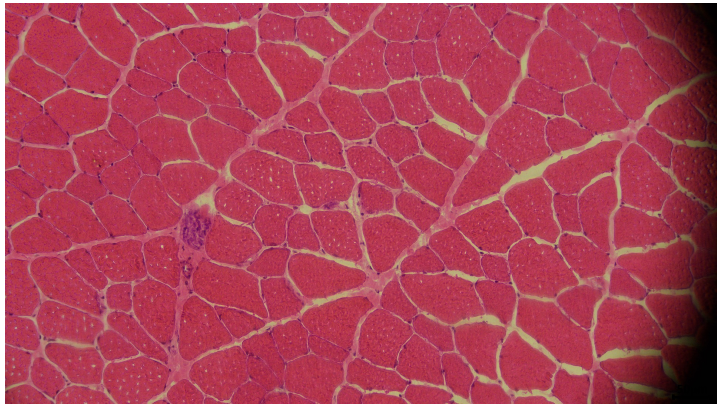

← 100 μm

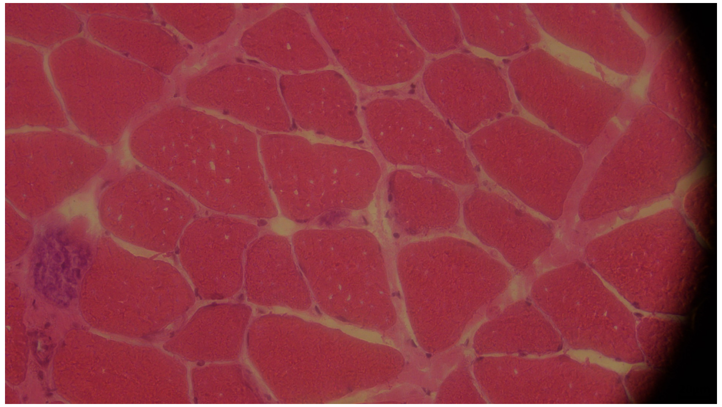

← 50 μm

# Part 3. Myosin ATPase  staining figures

DAPA+RT

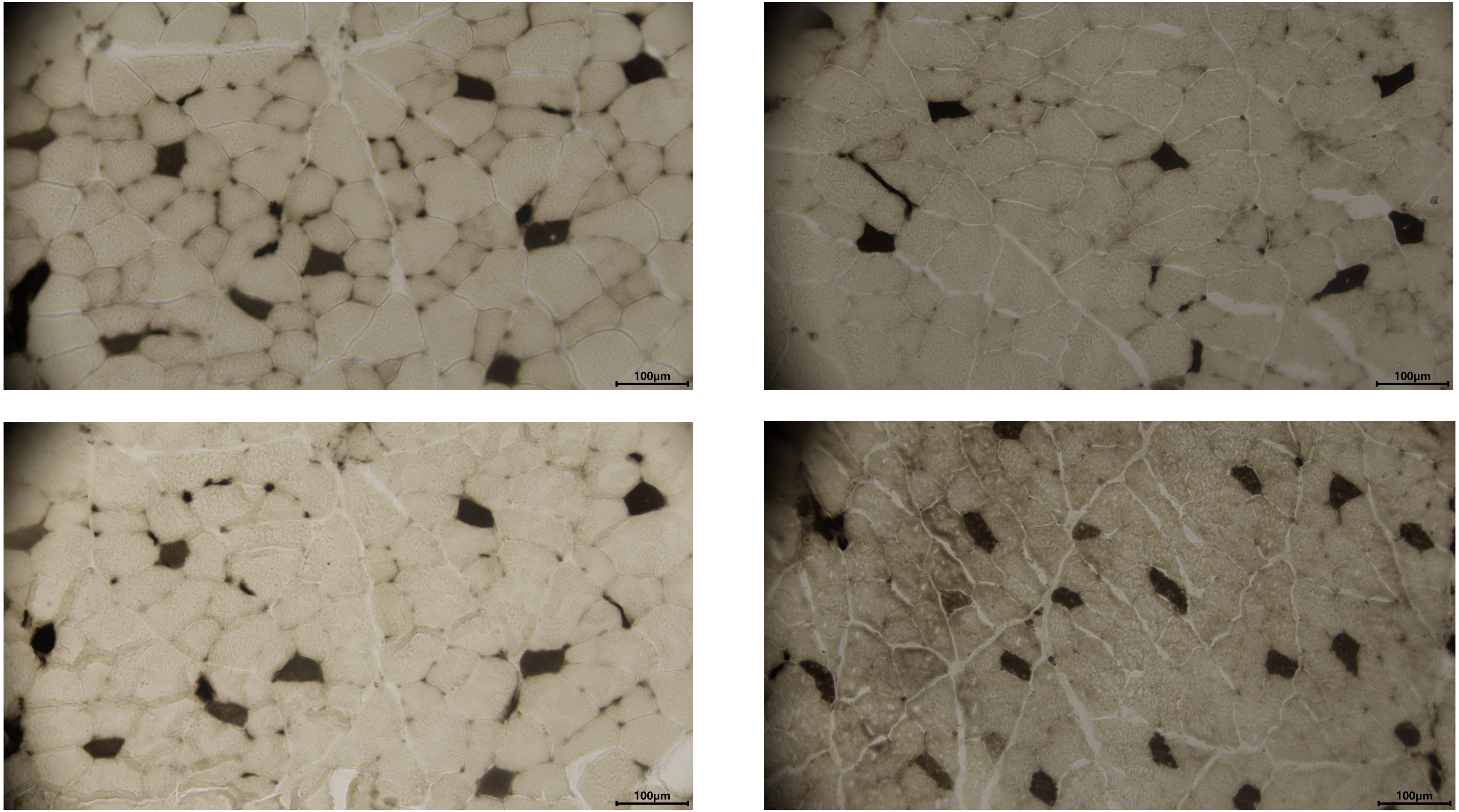

# DAPA+AE

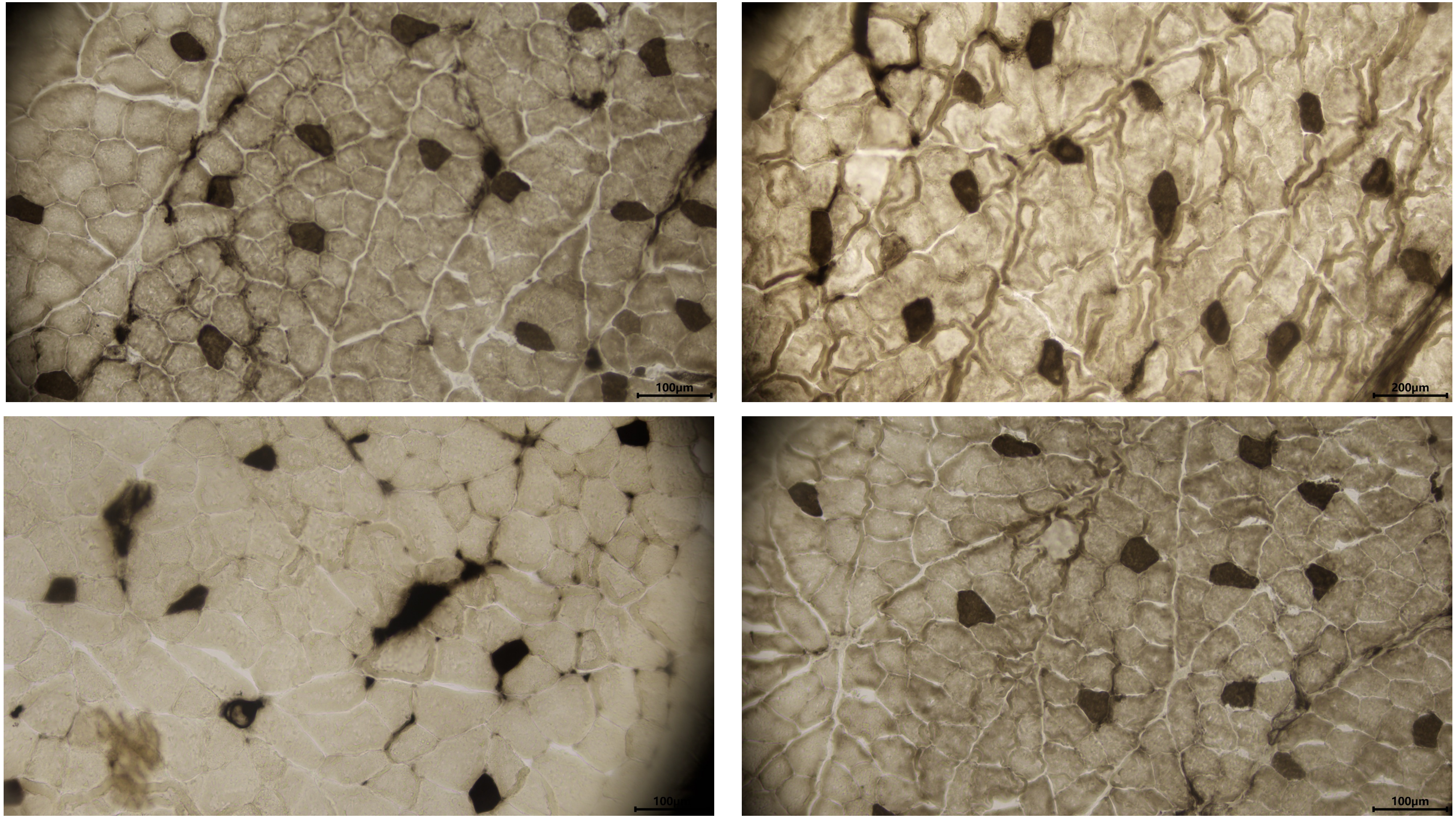

# DAPA

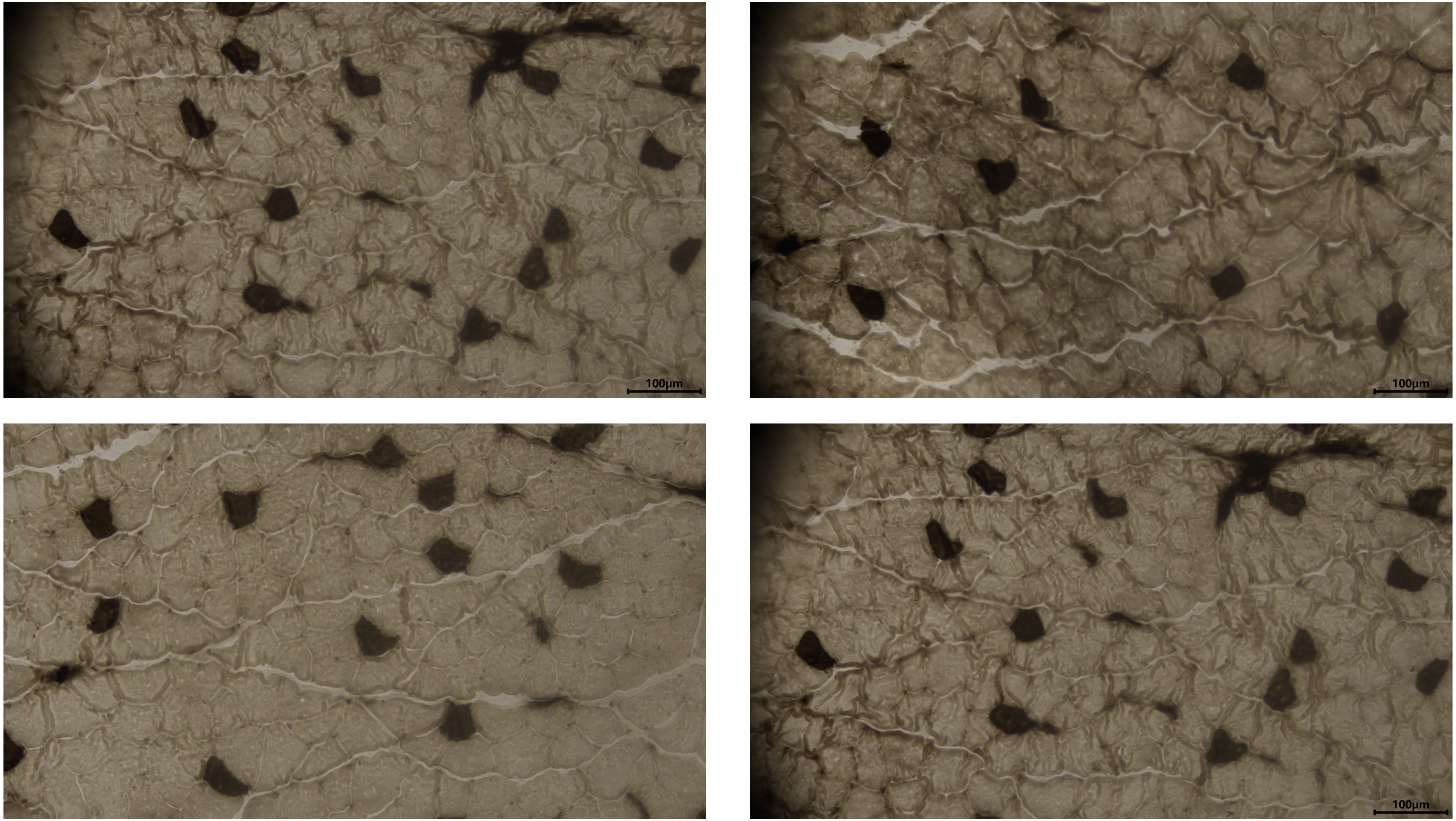

# CON

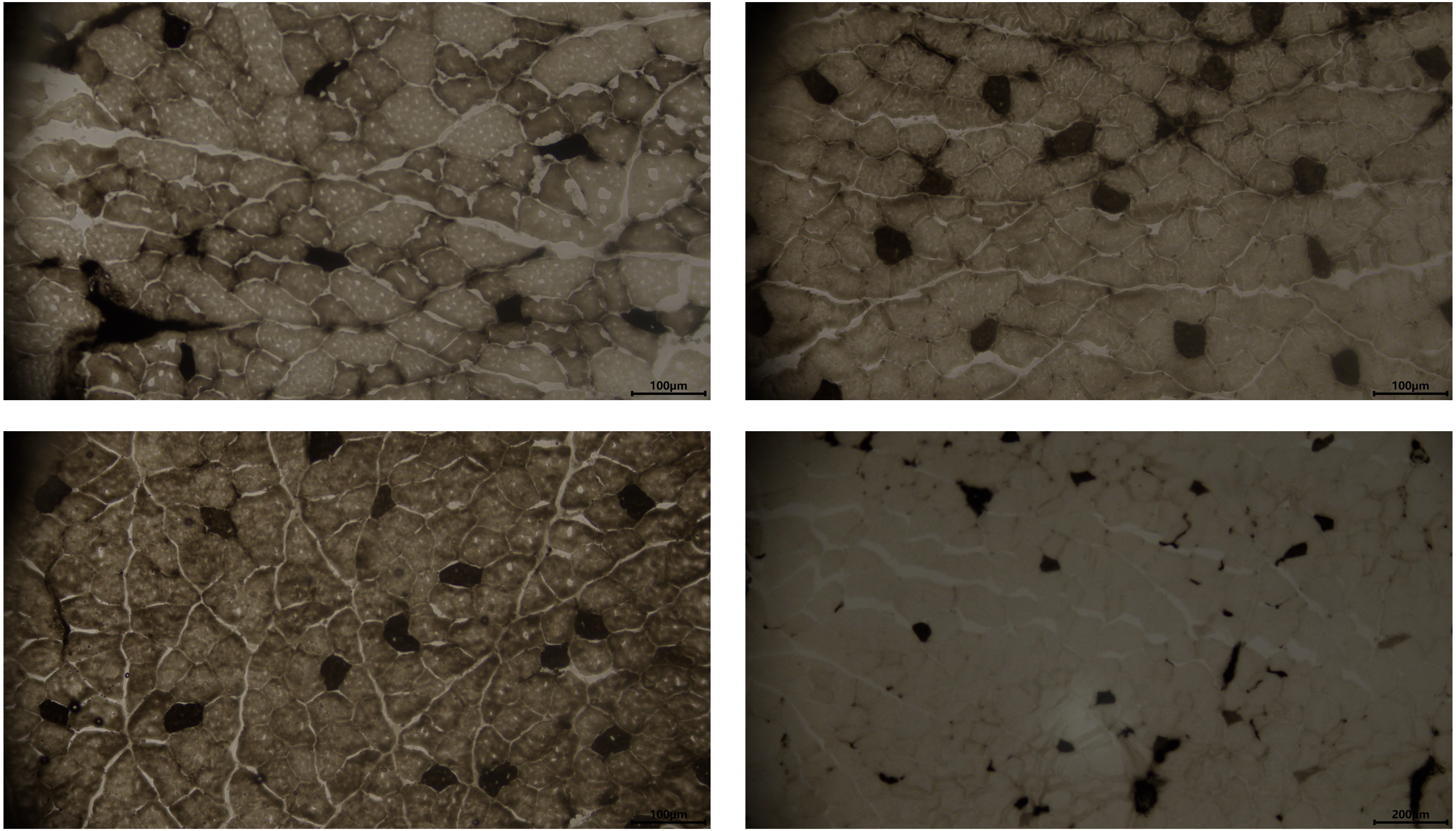

Supplement: Supplementary file 1 — Supplementary Material 1 [file 13098_2023_1130_MOESM1_ESM.pdf]
